# Vitamin D Deficiency and COVID-19: A Biological Database Study on Pathways and Gene-Disease Associations

**DOI:** 10.3390/ijms232214256

**Published:** 2022-11-17

**Authors:** Ángela Alcalá-Santiago, Miguel Rodríguez-Barranco, Marta Rava, María Ángeles Jiménez-Sousa, Ángel Gil, María José Sánchez, Esther Molina-Montes

**Affiliations:** 1 Department of Nutrition and Food Science, Faculty of Pharmacy, University of Granada, 18071 Granada, Spain; 2 Instituto de Investigación Biosanitaria ibs.GRANADA, 18012 Granada, Spain; 3 Institute of Nutrition and Food Technology (INYTA) ‘José Mataix’, Biomedical Research Centre, University of Granada, Avenida del Conocimiento s/n, 18071 Granada, Spain; 4 Andalusian School of Public Health, Cuesta del Observatorio 4, 18012 Granada, Spain; 5 CIBER de Epidemiología y Salud Pública (CIBERESP), 28029 Madrid, Spain; 6 National Center of Epidemiology (CNE), Institute of Health Carlos III (ISCIII), 28029 Madrid, Spain; 7 CIBER de Enfermedades Infecciosas (CIBERINFEC), 28029 Madrid, Spain; 8 Unit of Viral Infection and Immunity, National Center for Microbiology (CNM), Institute of Health Carlos III (ISCIII), 28029 Madrid, Spain; 9 Department of Biochemistry and Molecular Biology II, Faculty of Pharmacy, University of Granada, 18071 Granada, Spain; 10 CIBER de Obesidad y Nutrición (CIBEROBN), 28029 Madrid, Spain; 11 Department of Preventive Medicine and Public Health, Faculty of Medicine, University of Granada, 18011 Granada, Spain

**Keywords:** vitamin D, COVID-19, SARS-CoV-2, genes, nutrition, computational biology

## Abstract

Vitamin D (VD) is a fat-soluble vitamin, and pivotal for maintaining health. Several genetic markers have been related to a deficient VD status; these markers could confer an increased risk to develop osteoporosis and other chronic diseases. A VD deficiency could also be a determinant of a severe COVID-19 disease. This study aimed to interrogate genetic/biological databases on the biological implications of a VD deficiency and its association with diseases, to further explore its link with COVID-19. The genetic variants of both a VD deficiency and COVID-19 were identified in the genome-wide association studies (GWAS) catalog and other sources. We conducted enrichment analyses (considering corrected *p*-values < 0.05 as statistically significant) of the pathways, and gene-disease associations using tools, such as FUMA, REVIGO, DAVID and DisGeNET, and databases, such as the Kyoto Encyclopedia of Genes and Genomes (KEGG) and Gene Ontology (GO). There were 26 and 46 genes associated with a VD deficiency and COVID-19, respectively. However, there were no genes shared between the two. Genes related to a VD deficiency were involved in the metabolism of carbohydrates, retinol, drugs and xenobiotics, and were associated with the metabolic syndrome and related factors (obesity, hypertension and diabetes mellitus), as well as with neoplasms. There were few enriched pathways and disease connections for the COVID-19-related genes, among which some of the aforementioned comorbidities were also present. In conclusion, genetic factors that influence the VD levels in the body are most prominently associated with nutritional and metabolic diseases. A VD deficiency in high-risk populations could be therefore relevant in a severe COVID-19, underlining the need to examine whether a VD supplementation could reduce the severity of this disease.

## 1. Introduction

Vitamin D (VD) is a fat-soluble vitamin with multiple functions, the most important is bone mineralization by regulating the levels of calcium and phosphorus in the bone matrix. A VD deficiency is therefore related to bone deformation (rickets) or pain (osteomalacia) [1,2]. This deficiency can be caused by an insufficient diet supply and a low sun exposure, but also by the inadequate absorption of VD from the diet, or abnormal conversion of VD to its bioactive metabolites [3]. It is well-known that there are inter-individual differences in the absorption of VD from the diet that depend on the molecular forms of VD (whether it is vitamin D3 from animal sources or vitamin D2 from plant-based foods) [4], the type of food (the content of fats, dietary fiber, etc.) [4,5], or the chemical modifications of VD in supplements and fortified foods [6]. Furthermore, other endogenous factors, such as age [7], obesity [8], diseases that imply gastrointestinal or renal disorders [9] and genetic variations [5,8] also define the individual’s VD status. Regarding the latter, it has been estimated that genetic variants (including single nucleotide polymorphisms, SNPs) associated with VD explain about 20% of the heritability to the serum levels of this vitamin [10]. Thus, there are several risk factors of VD deficiency, among which the genetic factors seem to be key. Indeed, there have been identified over 60 genetic loci that seem to influence the VD levels in the blood, i.e., the bioavailable part of VD [10,11]. Fewer genetic variants seem to be strictly related to a VD deficiency in the population, according to recent genome-wide association studies (GWAS) that sought the genetic determinants of this phenotype [12].

VD levels of less than 20 ng/mL in serum indicate a VD deficiency [13]. VD is measured in the form of 25-hydroxyvitamin D 25(OH)D, also known as calcidiol, which reflects VD produced endogenously, as well as that obtained from foods and supplements. Moreover, this regards the most active metabolite of VD [14]. While dietary reference intakes (DRIs) have been established for VD, to prevent its deficiency [15], there is still scant evidence to conclude that adequate intake levels of this vitamin prevent potential adverse health outcomes other than bone health [16]. Despite the absence of information, in recent years there have been plenty of studies on the beneficial role of VD in cancer [17], cardiovascular disease (CVD) [18], diabetes mellitus [19] and obesity [20], amongst other diseases. Moreover, low VD levels have been related to a higher prevalence of comorbidities in the clinical setting [21]. Together, these studies have shown that VD has many biological functions beyond regulating the skeletal homeostasis [22]. In fact, other well-recognized actions of VD are related to cell growth, inflammatory processes, and the immune response in the body [2]. Due to its interaction with the immune system, modulating both the innate and adaptive immune systems, it has become more evident that VD is also involved in infectious diseases triggered by viruses, such as the human immunodeficiency virus (HIV) and the severe acute respiratory syndrome coronavirus 2 (SARS-CoV-2) that causes the COVID-19 disease [23,24].

The COVID-19 disease has been of global concern since the proclamation of this pandemic by the WHO [25]. COVID-19 causes diseases including, but not restricted to, respiratory and non-respiratory diseases, e.g., neurological complications and cardiovascular diseases, and conditions related to hypertensive disorders, amongst others [26,27,28]. Its pathogenesis has been elucidated to a great extent and several risk factors of this disease have been proposed, including the A type blood group, the presence of comorbidities and some genetic factors [29,30]. For instance, the variant rs17047200 in the TLL-1 gene seems to confer a higher risk of a severe COVID-19 disease [31,32,33] Regarding the comorbidities, certain diseases in COVID-19 patients are extremely worrying since deleterious effects have been observed in multimorbid patients, if infected by the virus [34,35,36]. Indeed, the prevalence of comorbidities is relatively high among those who develop severe COVID-19 [37,38,39]. Interestingly, some studies have also shown that COVID-19 patients tend to have deficient VD levels and a worse disease progression [39,40,41].

Nowadays, there are various biological databases that constitute large repositories on genes and associated diseases, and cellular, molecular and metabolic pathways linking genes with diseases. These tools allow for conducting bioinformatics studies by integrating and analyzing data from different sources, thereby facilitating the interpretation and visualization of the results. To the best of our knowledge, an attempt has been made to apply such tools to investigate the connection between comorbidities in the COVID-19 disease [42]. However, the role of VD was not accounted for in this study.

Thus, the objective of the current study was to interrogate the biological databases on the potential gene-disease associations between genetic factors related to a VD deficiency and the comorbid conditions and to shed light on the underlying mechanisms between VD and the comorbidities in the COVID-19 disease. We hypothesized that a susceptibility to a VD deficiency and/or to COVID-19, could increase the risk to develop more severe and fatal COVID-19 outcomes, given that comorbidities are common to both.

## 2. Results

### 2.1. Description of Genetic Variants of a VD Deficiency and COVID-19

The 20 SNPs associated with a VD deficiency, at a *p*-value threshold of 5 × 10^−8^, were annotated to 26 genes. Some variants were allocated in several genes of the UDP Glucuronosyltransferase Family 1 and 2. The selected variants and their corresponding genes are listed in Table 1.

Some genes to highlight are *GC* (VD binding protein gene), *CALCB* (calcitonin related polypeptide beta gene), and *LIPC* (lipase C, hepatic type gene). Furthermore, the 26 genes were distributed among chromosomes 1, 2, 4, 8, 11, 12, 15, 19 and 20. Chromosomes 4 and 11 showed variants with a high degree of correlation (R^2^ > 0.8) between them, i.e., some genetic variants were in the linkage disequilibrium (LD). For instance, in chromosome 4 (Figure S1A) the rs2282679 and rs11723621 variants were in the LD. The variants rs4944958 and rs12785878, located in chromosome 11, were also in the LD (R^2^ = 0.987) (Figure S1B). Fourteen variants were intronic.

Concerning COVID-19, 57 genetic variants were obtained, which were annotated to 46 genes (Table S1). The genes *ACE2* (angiotensin converting enzyme 2 gene) and genes of the major histocompatibility complex (*HLA-DRB1*) were of particular interest. All of the 46 genes were distributed across 17 chromosomes. Some variants in chromosomes 3, 9, 19 and 21 were in the LD. In particular, in chromosome 3 (Figure S2), eleven pairs of variants with an R^2^ greater than 0.8 were found (for example, rs71325088/rs13078854, rs10490770/rs13078854, rs10490770/rs71325088). In chromosome 9 (Figure S3A), chromosome 19 (Figure S3B) and chromosome 21 (Figure S3C), the variant pairs rs912805253/rs657152; rs2109069/rs12610495, and rs13050728/rs17860115, respectively, were in the LD, too. Forty-one variants were intronic.

Complete information for both sets of genetic variants, regarding the relative allele frequencies (RAF) and effect sizes of the associations, are shown in Tables S2 and S3.

Interestingly, there were no overlapping genes between a VD deficiency and COVID-19.

### 2.2. Functional Analyses and Enrichment Analyses

The resources used for the functional and enrichment analyses (FUMA, REVIGO and DAVID), derived information on gene expression patterns and shared molecular functions between the genes, regarding both cellular/molecular functions and metabolic pathways.

**Figure 1:**
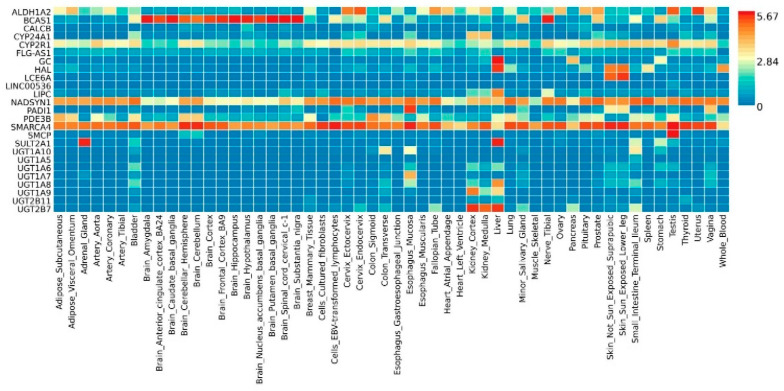
Heat map of the VD deficiency gene expression in different tissues. The tissue-specific expression patterns are based on GTEx v6 RNA−seq data. Cells in red represent a higher expression, compared to the cells in blue. Gene expression comparisons between tissues (horizontal comparison) within a gene (y axis) are comparable but not those of different genes within a tissue (vertical comparison). Thus, cells in red represent a higher expression of genes in a corresponding tissue, compared to other tissues, but do not represent a higher expression, compared to other genes. Source: FUMA.

For the VD deficiency genes, the heat map illustrating the expression profiles by genes and tissues (Figure 1) showed that the genes *SMARCA4, PDE3B, NADSYN1, ALDH1A2* and *CYP2R1* were highly expressed in adipose tissue, whereas *SMARCA4, NADSYN1* and, the *CYP2R1* genes were expressed in tissues of the circulatory system, especially in arteries, and in tissues of the immune system. Furthermore, the genes *GC, HAL, LIPC, SULT2A1* and *UGT2B7, UGT1A8* and *UGT1A9* showed a notable expression in the liver. In the lung, the genes SMARCA4 and NADSYN1 showed a relatively high expression. Likewise, for the COVID-19 genes, the expression profiles in the different tissues (Figure S4) showed that the *HLA-DRB1, LAMP2* and *PLSCR1* genes were more expressed in the adipose tissues; *HLA-DRB1, LAMP2, PLEKHA4, PLSCR1, RAVER1, TES* and *THBS3* genes were more represented in tissues of the circulatory system, especially in arteries and *HLA-DRB1, HLA-DQA1, IFNAR2, OAS1, RAVER1, TES* and *TYK2* genes were more represented in tissues of the immune system.

**Figure 2:**
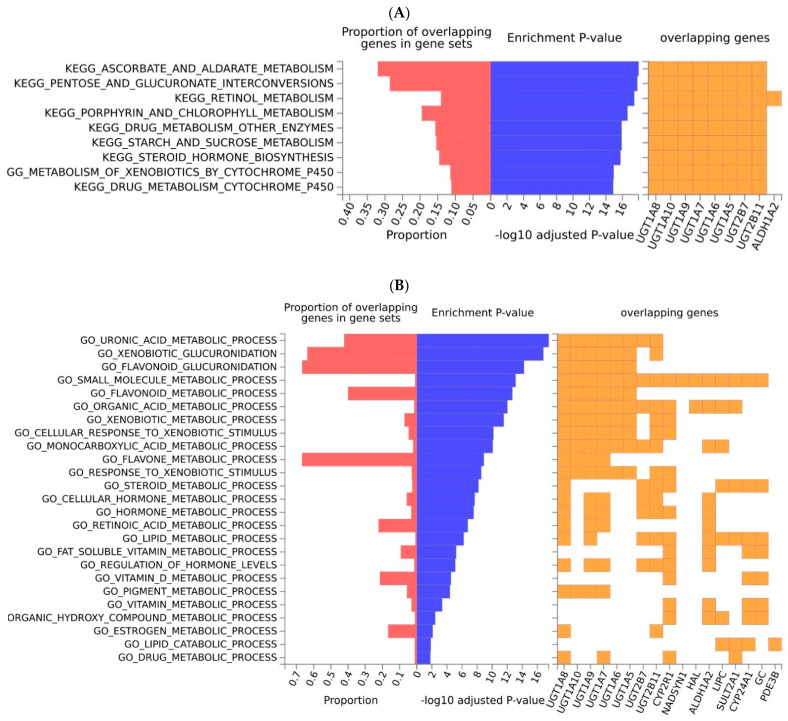
Enrichment analysis of a VD deficiency, regarding the KEGG pathways (**A**) and GO in MSigDB on the biological processes (**B**). Pathways and processes that are overrepresented in the gene set of interest are shown. Furthermore, only genes present in a given pathway or process are given. The overrepresentation of the gene set of interest (input genes) regarding pathways and functions was tested using the hypergeometric test against the pre−defined gene sets (background genes), obtained from MsigDB or KEGG. Input genes that are overlapping in the pathway or process, the enrichment *p*−value and the proportion of the overlapping genes (input genes relative to the tested gene set) are shown. Source: FUMA.

The results on the enrichment analyses performed on the Kyoto Encyclopedia of Genes and Genomes (KEGG) and Gene ontology (GO) databases, for the genes associated with a VD deficiency, are shown in Figure 2. The enrichment analyses included all of the genes associated with a VD deficiency and showed that some metabolic pathways were overrepresented (Figure 2A). In particular, it turned out that those related to the metabolism of sugars and carbohydrates (e.g., the ascorbate and aldarate metabolism using KEGG) were enriched with respect to all background genes. In fact, the genes of the UDP Glucuronosyltransferase Family 1 and Family 2 explained 30% of the genes present in this pathway. These genes were also enriched in the retinol metabolism pathway, together with gene *ALDH1A2,* as well as in the pathways related to the cytochrome P450 system. A total number of 17 genes were also involved in 25 biological processes, according to the GO pathways (Figure 2B), including the uronic acid metabolic processes, and the metabolic processes related to xenobiotics, drugs, steroids and other hormones, as well as lipids and flavonoids. The molecular functions in MsigDB were related to these processes, too; for example, the glucuronosyltransferase activity emerged as the most important function (data not shown). The Reactome database showed an enrichment for glucuronidation, the VD metabolism, the metabolism of lipids and steroids, and biological oxidations (data not shown). Similarly, in WikiPathways we observed an enrichment for glucuronidation, the VD metabolism, the oxidation by cytochrome P450 and the pathways related to nuclear receptors and the transcription factors NRF2. Some of these processes were semantically similar to the GO terms in REVIGO, namely the metabolic processes of vitamins, steroids, flavonoids, lipids and xenobiotics (Figure S5A).

**Figure 3:**
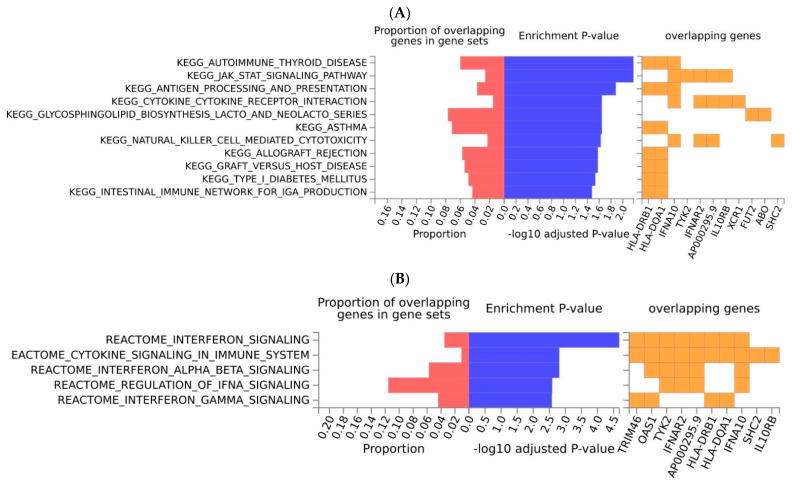
Enrichment analysis of COVID-19, regarding the KEGG pathways (**A**) and signaling pathways in the Reactome database (**B**). Pathways that are overrepresented in the gene set of interest are shown. Furthermore, only genes present in a given pathway are given. Overrepresentation of the gene set of interest (input genes), regarding the pathways was tested using the hypergeometric test against the pre−defined gene sets (background genes) obtained from Reactome or KEGG. Input genes that are overlapping in the pathway, the enrichment *p*-value and the proportion of the overlapping genes (input genes relative to the tested gene set) are shown. Source: FUMA.

Regarding COVID-19 (Figure 3), we observed that the selected genes were involved in the immunological processes, such as antigen processing and presentation pathways, and immune regulation pathways (e.g., of natural killer cells, and cytokines). Among them, pathways related to autoimmune diseases and asthma were enriched in the KEGG database (Figure 3A). Enriched signaling pathways were also restricted to the immunological processes (Figure 3B). The *HLA* genes (the gene family of the major histocompatibility complex, *MHC*), together with the *IL10RB* and *IFNA10* genes, mainly, were driving these pathways. Of note, no molecular function was enriched in the GO database. The pathway maps of COVID-19 genes showed interactions between these genes and cytokines, as well as processes that comprise the immune recognition against viral infections (Figure S6). In addition, in the MsigDB the biological processes and immunologic signatures that appeared significantly enriched, were also linked to pathways mediated by cytokines and other immunologic cells (for example, CD4 T cells and macrophages). There were two main semantically similar GO terms: negative regulation of the viral genomes and receptor signaling pathways (Figure S5B).

### 2.3. Gene-Disease Associations

The platforms and tools used for the gene-disease association analyses (DisGeNET and ClusterProfiler) produced gene-disease clusters, networks and heat maps by diseases and groups of diseases. Results on the variant-disease associations are not shown for the sake of clarity and because the results were fairly similar.

**Figure 4:**
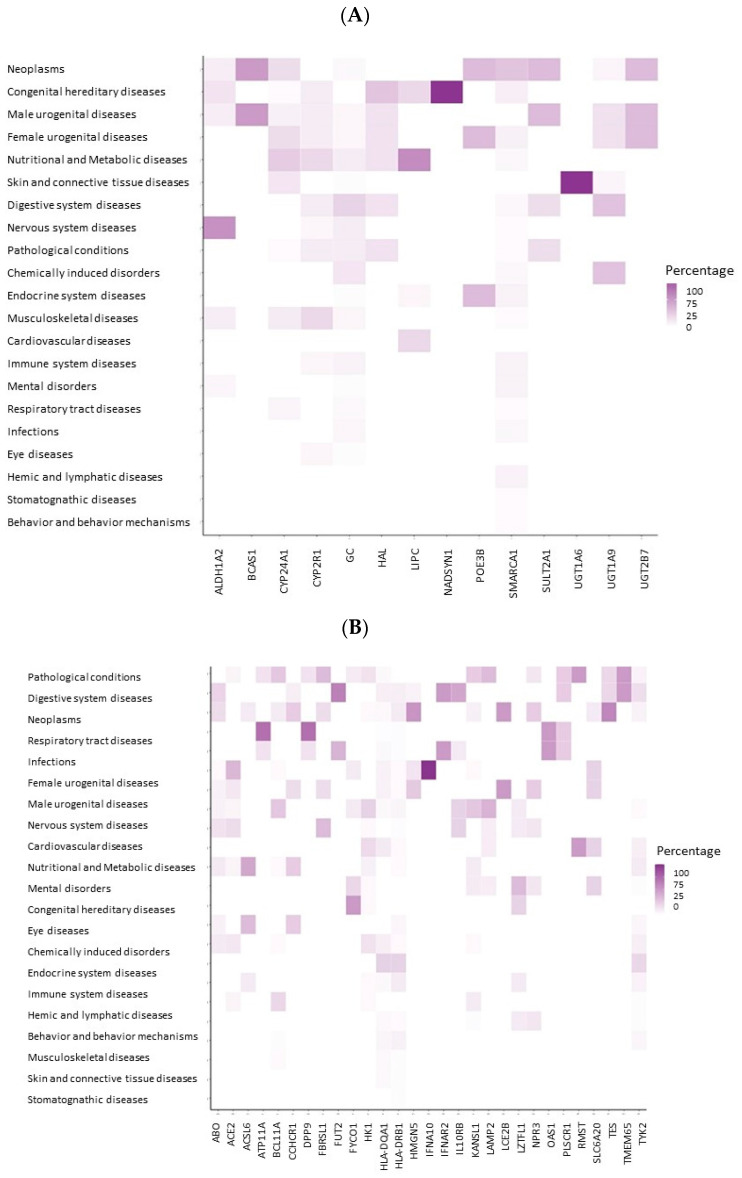
Gene-disease heat map by groups of diseases for VD deficiency genes (**A**) and COVID-19 (**B**). The x-scale shows the list of genes (14 for VD and 29 for COVID-19); the y-axis shows the diseases grouped by the MeSH disease classes. The color scale is proportional to the percentage of diseases in each disease class. Source: DisGeNET.

For a VD deficiency, there were 14 genes matching gene IDs in the DisGeNET database: *CYP2R1, CYP24A1, NADSYN1, HAL, ALDH1A2, LIPC, SMARCA4, SULT2A1, BCAS1, UGT1A9, UGT1A6, UGT2B7, GC* and *PDE3B*. The gene-disease networks of these genes (Figure S7), showed that there were strong interactions between the genes *GC*, *ALDH1A2* and the Cytochrome P450 Family 2 genes, with several chronic diseases, including neurodegenerative diseases, cardiovascular diseases, musculoskeletal diseases, renal kidney diseases and various cancer types. Separate clusters were seen for the remaining genes. For instance, the gene *LIPC* formed a cluster of metabolic diseases. The gene-disease heat maps (Figure S8) and gene-disease heat maps by groups of diseases (Figure 4A) provided a deeper insight into these associations, revealing that genes of a VD deficiency have been mostly associated with diseases, such as diabetes and insulin resistance (Figures S7 and S8).

By the disease groups, a higher scoring (strength of association) was noted for neoplasms, congenital diseases (mostly driven by the *NADSYN1* gene), male and female urogenital diseases and nutritional/metabolic diseases. Other diseases received a lower score. Importantly, the *LIPC* gene was more prominently associated with nutritional/metabolic diseases, with the genes *GC, HAL* and the Cytochrome P450 Family also being implicated.

For COVID-19, 29 genes were available in the DisGeNET database. Networks (Figure S9) and heatmaps of gene-disease associations, at the individual level (Figure S10) and by groups of diseases (Figure 4B) showed that: the five top gene-disease associations were congenital diseases, digestive system diseases, neoplasms, respiratory tract diseases and infections. Among the genes associated with respiratory tract diseases, the most significant ones were *ATP11A* and *DPP9*. Less importantly, though, were urogenital, nervous system and cardiovascular diseases. Associations with nutritional and metabolic diseases were also less important, and were mainly linked to the genes *RMST, HK1, HLA-DQA1* and *LAMP2*. In diseases of the immune system, there were also highly relevant genes, such as *HLA-DQA1, HLA-DRB1* and *TYK*. In cardiovascular diseases, the genes *FBRSL1, IL10RB, ACE2* and *ABO* were those that were outstanding (Figure S10).

The results on the enrichment analysis for the genes associated with a VD deficiency are shown in Table 2. This analysis revealed the diseases in which the studied genes were more enriched, that is, more overrepresented. VD deficiency genes were firstly overrepresented in VD-related conditions (VD status and rickets). In addition, these genes were enriched for some metabolic diseases (for example, metabolic syndrome; corrected *p*-value = 2.933 × 10^−5^ and cardiovascular diseases (coronary heart disease; corrected *p*-value = 1.732 × 10^−4^).

The results on the enrichment analysis for the genes associated with COVID-19 are shown in Table 3. COVID-19-related genes were more enriched in conditions of the liver or respiratory tract (such as coughing and rales), and to a certain degree with autoimmune-related diseases.

**Figure 5:**
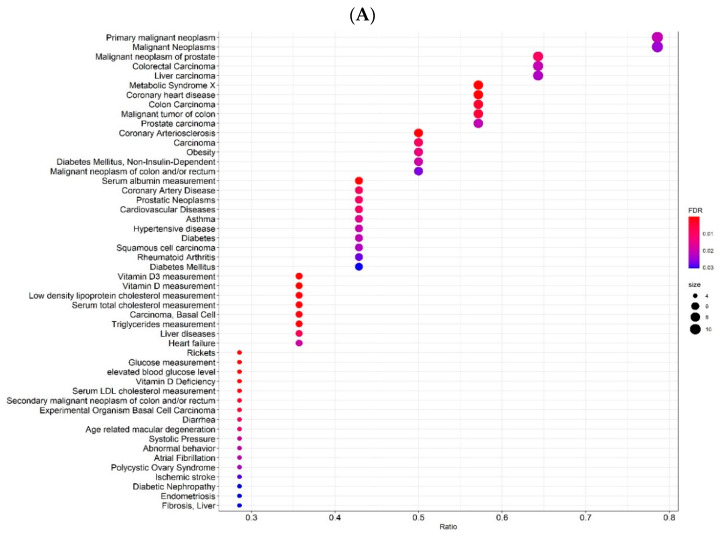

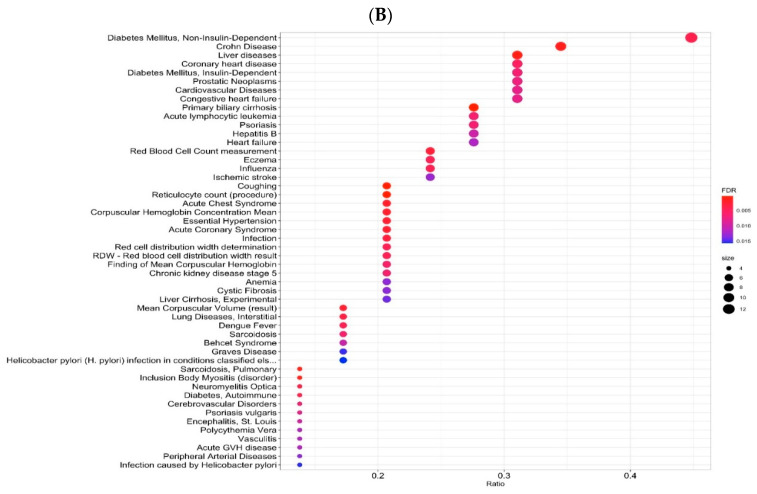
Enrichment Analysis of the genes associated with a VD deficiency (**A**) and COVID-19 (**B**). Representation of the existing association between the diseases and the enriched genes, based on the ratio (input genes relative/associated) and the FDR. The size of the dots refers to the number of genes enriched for that disease (the greater the number of genes, the larger the dot). The FDR is defined by a color scale, the closer it is to red, the greater the FDR value and the greater the association.

Using the ClusterProfiler tool, the enrichment analysis was extended further to 50 diseases. The enrichment plots are shown in Figure 5. Several cancer types, metabolic syndrome and its components (such as obesity, diabetes, hypertension) emerged first. VD deficiency genes were highly represented in these diseases (Ratio > 0.5). Interestingly, some of these diseases (diabetes mellitus, coronary heart disease and cardiovascular diseases) were also significantly enriched (corrected *p*-value < 0.05, and Ratio > 0.3) for COVID-19-associated genes. Thus, there were common comorbid conditions enriched in genes associated with both a VD deficiency and COVID-19. Some viral infections and respiratory tract diseases were also apparent, the number of enriched genes was also high for these diseases in COVID-19.

## 3. Discussion

In this study, using several biological databases and tools, we have analyzed whether genes associated with a VD deficiency and the COVID-19 disease were interrelated, and whether molecular, cellular and metabolic pathways and diseases connected to these genes are shared between the two. Whereas there were no common genes, this study shows that the genes associated with a VD deficiency were mainly involved in the biological processes and pathways related to the metabolism of carbohydrates, retinol, steroids, drugs and xenobiotics. A VD deficiency is also more prominently associated with nutritional and metabolic diseases, all being comorbid conditions in the COVID-19 disease. Likely, genes associated with this illness tended to be linked to similar diseases, suggesting that a genetic predisposition to a VD deficiency might have a significant impact on COVID-19-related outcomes. Gene ontologies and pathways for COVID-19 were more predominantly related to natural killer cells and the production of cytokines, as well as to other biological processes implicated in the inflammatory and immune responses that are activated by this disease.

An earlier bioinformatics study on the potential genes associated with COVID-19 and comorbidities (hypertension, diabetes mellitus and coronary artery disease) found that there were eleven shared genes (*TLR4, NLRP3, MBL2, IL6, IL1RN, IL1B, CX3CR1, CCR5, AGT, ACE* and *F2*) [42]. These genes were enriched in nearly 300 biological pathways and 15 molecular functions, all of which are related to immune and inflammatory processes, which reinforces the importance of these mechanisms in the COVID-19 disease. Likewise, the authors showed that the three comorbidities were also connected to metabolic and immune system alterations. In our study, we did not consider genes associated with commonly present comorbidities in COVID-19, since our focus was put on a VD deficiency. Furthermore, it is important to highlight that, unlike this previous study, we kept in the analyses, only the genetic variants that met the *p*-value threshold of the GWAS studies, to retain the true positives, i.e., those robustly associated variants with the trait of interest. As generally known, to account for multiple testing in the GWAS studies, it is convenient to keep a stricter *p*-value threshold [43]. Despite this issue, our results also support that the SNPs associated with COVID-19 were implicated in the immune system function and inflammation-related diseases.

Among the best-established genetic variants in the COVID-19 disease, there are those of the gene *ACE*. This gene encodes the angiotensin-converting enzyme in the renin-angiotensin-aldosterone system (RAAS), which is involved in the blood pressure regulation and electrolyte balance, and possibly also in the pathophysiology of COVID-19 [44]. Genetic variants of the *ACE* gene affecting the treatment response of ACE inhibitors have been also described [45]. Moreover, exacerbation of symptoms in COVID-19 has been associated with a genetic predisposition to severe illness via this gene in multiple studies [46,47]. According to these studies, the alterations in the expression of the *ACE* gene lead to the accumulation of angiotensin, which seem to promote the infection of SARS-CoV-2 [46,48]. It is important to highlight that VD plays a central role in the RAAS, since it regulates the expression and production of renin [49]. The fact that VD and the RAAS are connected might explain why VD deficiency genes were enriched in diseases including but not limited to hypertension and coronary heart disease, in our study. Other COVID-19-related genes that were implicated in nutritional/metabolic diseases and other comorbid conditions (the *ABO* gene and others) seem to have no clear relationship with the metabolism and actions of VD [50]. The expression of the immune system-related SNPs in COVID-19 (the *IFNA, IL10* and *HLA* genes), however, could be affected by the levels of VD. High levels of VD are known to suppress the B cell and T cell proliferation, the monocyte production of inflammatory cytokines (interleukins and tumor necrosis factor-alpha, TNF-α) and the expression of the MHC class II molecules [51]. Thus, given the importance of VD in the modulation of the immune function, it has been proposed that VD could have a positive impact on the COVID-19 disease [52,53]. In the last few years, various systematic reviews and meta-analyses have updated the evidence on the potential effects of a VD supplementation in patients diagnosed with COVID-19 in the clinical setting [54,55,56]. Furthermore, lower inflammatory markers have been observed in COVID-19 patients treated with VD [57]. However, not all studies support that VD supplementation is an effective treatment to reduce the risk of severe COVID-19 and its mortality. Therefore, there is still no solid evidence on this issue [56]. Nevertheless, a profound VD deficiency has been observed in patients with a severe COVID-19 illness [40,41], supporting that VD could reduce the harmful effects of the SarsCov-2 infection. As far as we are aware, whether these COVID-19 patients present a genetic susceptibility to a VD deficiency, has been not investigated.

Regarding a VD deficiency, the genes *LIPC*, *HAL, GC* and the Cytochrome P450 Family genes were among those which are more strongly associated with nutritional/metabolic diseases. The *LIPC* gene encodes the hepatic triglyceride lipase, which is expressed in the liver. It catalyzes the hydrolysis of triglycerides and phospholipids present in the circulating plasma. Its mutation increases the likelihood to develop dyslipidemia, hypertension and insulin resistance, and in turn, the metabolic syndrome [58]. Carriers of the risk alleles of the *GC* gene have been also associated with a higher risk of developing this syndrome and its components in various study populations [59,60]. A variation in the *GC* gene has also been associated with obesity [61]. As we have shown, this gene is prominently expressed in the adipose tissue, where VD can also regulate the biological processes including adipogenesis, inflammation, oxidative stress and metabolism in mature adipocytes [61]. Accordingly, low VD levels can lead to obesity, this is another component of the metabolic syndrome and an important cardiovascular disease risk factor [22,61]. As for the other genes, there is less evidence supporting their role in the metabolic syndrome or related components. Overall, however, all of these VD deficiency genes have not been linked to COVID-19, in any study. Thus, while there is no a common genetic background between VD and COVID-19, our results support that it is plausible that a VD deficiency causes comorbid conditions, posing a higher risk to develop a severe COVID-19 disease. In fact, as we have already mentioned, the prevalence of comorbidities in hospitalized patients with a severe COVID-19 is high [34]. VD could be key to treat the COVID-19 disease in these patients [5]. For instance, VD has been shown to improve the prognosis of COVID-19 in patients with renal kidney disease, which is also determined by the metabolic syndrome risk factors [62].

There are some limitations of this study. Due to a lack of matching gene IDs in some databases, because their associations are still uncertain in these genetic/biological data repositories or collections, we were not able to evaluate the connections with other potential pathways and diseases. Furthermore, we did not consider the gene-environment interactions or the potential confounders, such as smoking, in our analyses, which might have affected the results obtained. However, to our knowledge, this issue is not accounted for in any of these resources. Moreover, our search of genetic variants was restricted to a VD deficiency; those for which a VD deficiency was not the reported trait, were removed from the analyses. This way, the genetic variants that have been associated with the VD levels in blood in the GWAS studies, but not strictly with a VD deficiency, were not included. For example, the gene *VDR*, which encodes for the VD receptor (VDR), is activated by 1,25(OH)D and forms a heterodimer with the retinoid X receptor to regulate the transcription of other genes. The VDR has been found in virtually all cell types, which may explain its multiple and novel actions of VD on different tissues. Indeed evidence of the extraskeletal effects of VD, including xenobiotic detoxification, oxidative stress reduction, neuroprotective functions, antimicrobial defense, immunoregulation, anti-inflammatory/anticancer actions and cardiovascular benefits, is mediated by VDR [22]. Importantly, the expression of VDR depends on the levels of VD; the expression is only decreased when the VD levels are deficient [63]. Therefore, polymorphisms of the *VDR* gene were not included in this study. To check the extent to which this gene could influence our results, we carried out a secondary analysis involving all genes, including the *VDR* gene, where we observed that the genetic variants of the latter were overrepresented in the bone-related diseases (rheumatoid arthritis, rickets and osteoporosis), in various neoplasms and hypertensive disease. Furthermore, with a similar intensity to the *GC* gene, the gene *VDR* seemed to be involved in nutritional and metabolic diseases. Moreover, the same diseases came up in the disease-enrichment analyses and similar processes emerged in other functional analyses (Figure S11). Thus, not having considered the VDR genetic variants should not have materially affected our results.

Concerning the strengths of this study, we have used several resources and web-based tools to research the relationship between SNPs, genes, molecular functions, pathways and diseases. The use of several tools has enabled us to confirm the findings and to enhance the interpretations and our understanding on these relations. Finally, we evaluated whether proxy SNPs, i.e., SNPs in the LD among the selected genes and chromosomes, had any redundancy in our variant-disease analyses, possibly affecting multiple testing corrections in the enrichment analyses. As aforementioned, there were few SNP pairs in the LD; removing those SNPs did not alter the results. Furthermore, the enrichment and pathway analyses were undertaken on all genetic variants (and annotated genes), regardless of the population ancestry, since we included both those of European and non-European ancestry. It is unlikely that this issue has affected our results, given that the molecular functions, pathways and disease phenotypes are overall highly conserved [64]. Finally, we set the *p*-value threshold to 5 × 10^−8^, to keep only the true associations between the genetic variants with a VD deficiency and COVID-19 for the analyses.

## 4. Materials and Methods

### 4.1. Selection of Genetic Variants

We used two databases to identify SNPs related to a VD Deficiency and COVID-19: the GWAS catalog (https://www.ebi.ac.uk/gwas/ (accessed on 21 June 2022) and PhenoScanner (http://www.phenoscanner.medschl.cam.ac.uk/ (accessed on 21 June 2022)) database.

Both the GWAS catalog and PhenoScanner offer a high-quality curated collection of all published GWAS studies [65]. Specifically, the goal of these resources is to facilitate the cross-reference of genetic variants with a wide range of phenotypes. For example, PhenoScanner contains more than 65 billion associations and more than 150 million unique genetic variants [66,67]. We sought in both genomic databases, the SNPs associated with a VD deficiency or COVID-19 with a *p*-value lower than 5 × 10^−8^.

We extracted 20 SNPs (as of June 2022) associated with a VD deficiency, which came from seven GWAS studies. Of this set of SNPs, 17 SNPs were from populations with European ancestry and three SNPs were from Asian populations.

Regarding COVID-19, we applied the same steps. In this case, the number of selected variants was 57 (as of August 2022). Likewise, the majority of those SNPs were identified in the European GWAS studies.

### 4.2. Annotation of Genes

We used the GWAS catalog [65] and Ensembl [68,69] to annotate the SNPs previously identified to their genes. The information related to the variants of interest was extracted from the GWAS catalog. Indeed, the GWAS catalog database provides information about the genes to which the variants of interest are associated, the chromosome and its position, amongst the other data of interest. Ensemble (https://www.ensembl.org/index.html (accessed on 3 November 2022)) was used to complement this information via the variant effect predictor (VEP) application (https://www.ensembl.org/info/docs/tools/vep/index.html accessed on 22 June 2022) [69], and GeneCards (https://www.genecards.org/ (accessed on 22 June 2022)) was used to complete the name of each gene. These tools allowed us to retrieve information on the location of the variant and on the consequences of the variation in the DNA sequence (whether the variant is intronic, missense, etc.). The GRCh38.p13 genome was used as a reference for the gene annotations.

The same procedure was carried out in both cases, for a VD deficiency and for COVID-19.

### 4.3. Linkage Disequilibrium Analysis

We subjected all variants to the LD analysis to explore the existence of closely related genetic variants within a chromosome [70]. Given that the alleles of such variants are inherited together more commonly than expected, they are assumed to be correlated; the higher the correlation (R2), the closer the variants [71]. These variants could therefore imply a redundancy in the analysis, if not accounted for. We used the LD matrix tool provided by LDLINK (National Cancer Institute, NHI). (https://ldlink.nci.nih.gov/?tab=ldmatrix (accessed on 22 June 2022)) to identify the genetic variants in the LD. An interactive heat map matrix of the pairwise LD statistics for each chromosome in which more than one variant associated with a VD deficiency or COVID-19 was present, was obtained.

### 4.4. Identification of the Overlapping Genes

The related genes between a VD deficiency and COVID-19 were cross-analyzed, using custom functions developed using R software version 4.1.2 [72]. More precisely, two lists of genes and variants, one with the VD deficiency genes and the other with the COVID-19 genes were crossed to identify the common variants and genes. Venn diagrams were created to further visualize the potential overlapping genes.

### 4.5. Biological Database Studies

We performed the functional and enrichment analyses on the list of genes and variants selected in the previous steps, using several tools and resources. The type of analysis and the tools used to carry out these analyses are described below.

#### 4.5.1. In Silico Functional Analyses and Enrichment Analyses

First, we run functional and enrichment analyses using DAVID, FUMA and REVIGO.

DAVID is a bioinformatics resource that enables the functional annotation and enrichment analysis of gene lists. This tool allows to agglomerate the gene identifiers from resources, such as Ensembl, Gene Ontology (GO) and the Kyoto Encyclopedia of Genes and Genomes (KEGG) [73]. Through DAVID (https://david.ncifcrf.gov/tools.jsp (accessed on 23 June 2022)) we gathered information on the diseases that are related to the selected genes and variants, the functional annotations and pathways.

With FUMA, using the GENE2FUNC module (https://fuma.ctglab.nl/gene2func (accessed on 11 July 2022)), we obtained information on the expression patterns of the genes and shared molecular functions between the genes. In particular, we obtained the gene expression heat maps and results on the functional analyses for a given molecular, cellular or metabolic pathway, and even the presence in certain diseases. Additionally, the expression of the genes of interest in different tissues (from Gtex v6 data) [74] were extracted to compare the expression profiles. FUMA is another web-based platform that can be used to prioritize, visualize and interpret the GWAS results [75]. Its main objective is to generate functional biological information on the GWAS hits. It also provides gene-based, pathway and tissue enrichment results. The platform is linked to popular resources, such as GO, WikiPathways and MsigDB.

In the enrichment analysis, hypergeometric tests were performed to check if the genes of interest are overrepresented in a predefined gene set. In our case, the latter gene set, i.e., the so-called background genes, were the “universe”. The gene sets were compared with the aforementioned background genes regarding the pathways and diseases using different data repositories (MsigDB, WikiPathways, GO and KEGG). GO divides the functions of genes into three parts: cellular component, molecular function, and biological process. KEGG provides information on the metabolic pathways and also annotates enzymes that catalyze each step of these pathways. The Molecular Signatures Database (MsigDB) contains a collection of canonical pathways derived from KEGG, Reactome, WikiPathways and others. The immunologic signature gene set of MsigDB was also interrogated via FUMA. All of these databases were accessed in July 2022.

REVIGO is a web platform that uses GO terms from previously conducted enrichment analyses for further visualization and interpretation of the results. It uses a clustering algorithm, based on semantic similarity measures, giving several outputs to help in the interpretation [76].

All GO terms were obtained from the enrichment analyses conducted on the genes of interest, using ClusterProfiler package in the R software [77,78]. These analyses were performed separately for the VD deficiency and COVID-19 genes, and by combining the two. In addition to the GO terms, we obtained the *p*-values that indicated the degree of association between the gene and the GO term.

In REVIGO (http://revigo.irb.hr/FAQ.aspx#q01 (accessed on 15 July 2022)), each GO term, together with its *p*-value, was introduced, to obtain the different graphs for the interpretation of the results (multidimensional scaling plots, tree maps and tag clouds).

#### 4.5.2. SNP-Disease and Gene-Disease Associations, including Enrichment Analyses

To explore the associations between the VD deficiency and COVID-19-associated genes, we used the DisGeNET database in R software (https://www.disgenet.org/disgenet2r (accessed on 3 November 2022)) from the disgenet2r package [79]. This tool allows to retrieve, visualize and expand the variant-disease and gene-disease associations.

The degree of association between a certain SNP and a disease, or a gene and a disease, is shown through a “score”. Indeed, the DisGeNET score ranks the gene-disease associations, according to their level of evidence calculated by an algorithm that considers the number and type of sources present in the database, and the number of publications that support the association [80,81].

The list of genes of interest was introduced as the HGNC symbols or Entrez gene identifiers. These results were represented as gene-disease heat maps; gene-class of diseases heat maps; or gene-disease networks. In this study, the DisGeNET curated database was used, which contains 9703 genes, 11,181 diseases and 84,038 associations (https://www.disgenet.org/dbinfo). This database was also accessed in 30 July 2022.

In addition, an enrichment analysis on the diseases in DisGeNET was performed for the same input of genes and the curated database available in DisGeNET. In the enrichment analysis, again, Fisher’s test was applied to all genes (universe; N = 9703) concerning the set of genes that were previously identified (VD deficiency and/or COVID-19). The *p*-values resulting from the multiple Fisher tests were corrected by the Benjamini–Hochberg (BH) method and for the false discovery rate using a FDR *p*-value of 0.05 [82]. The number of genes from the input list that were associated with the outcome reflects the BgRatio.

### 4.6. Workflow of the Analysis

**Figure 6:**
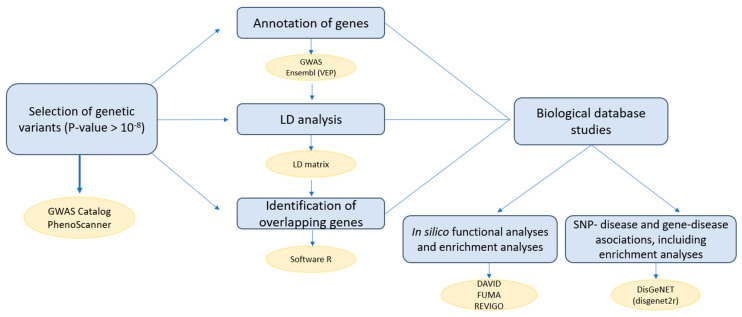
Workflow of the data analysis.

Figure 6 shows our workflow diagram, illustrating the different steps and tools used to carry out our study. In summary, we started the analysis by extracting the variants associated with a Vitamin D (VD) deficiency and COVID-19, from the GWAS catalog and PhenoScanner database (*p*-value threshold 5 × 10^−8^) [65,67]. Subsequently, we used the Variant Effect Prediction tool (VEP, in Ensembl) to annotate these variants to genes [68]. The linkage disequilibrium (LD) of the selected variants was examined [70,71], as well as the existence of the overlapping variants or genes. Then, a study of the biological databases was carried out; firstly, an in silico functional analysis and enrichment analysis (using the platforms DAVID [73], FUMA [75] and REVIGO [76]) was conducted, and secondly, a study of the SNP-disease and gene-disease associations, including enrichment analyses (using disgenet2r package and ClusterProfiler) was carried out.

## 5. Conclusions

In the current study, we have made use of the genetic variants of a VD deficiency and COVID-19 disease to explore the molecular functions, metabolic pathways, and diseases relevant to both a VD deficiency and COVID-19. Using several complementary approaches, we provide insight into the link between a genetic susceptibility to low levels of VD and to the COVID-19 disease. Specifically, our results support the hypothesis that a genetically predicted VD deficiency might imply a higher risk of developing comorbid conditions, and in turn, a severe or fatal COVID-19. Therefore, the role of VD in this context should be addressed in future studies to evaluate whether the supplementation of VD in the population susceptible to a VD deficiency could reduce the COVID-19 severity. Furthermore, molecular and functional studies are needed to evidence the molecular mechanisms by which a VD deficiency and the COVID-19 disease are connected. This knowledge is also crucial to implement efficient strategies to prevent and control this disease.

## Figures and Tables

**Table 1:** Genes and variants associated with a VD deficiency. Information on the genes to which these variants belong, the risk allele of each SNP, location in the genome (chromosome: chr) for the hg38 coordinates, and the *p*-value (existing degree of significance between that variant and the disease). Study accession numbers obtained from the GWAS catalog. Population: European (GCST000697, GCST90020244), Asian (GCST90101732).

Variant	Risk Allele	Gene	Location (Chr: Position)	*p*-Value	Study Accession	PubMed ID
rs3750297	A	PADI1	1:17233181	3 × 10^−10^	GCST90020244	34308111
rs12123821	T	FLG-AS1	1:152206676	6 × 10^−26^		
rs4845491	C	SMCP, LCE6A	1:152877093	7 × 10^−10^		
rs3755322	G	UGT1A9, UGT1A5, UGT1A10, UGT1A7, UGT1A8, UGT1A6	2:233713141	6 × 10^−12^		
rs6600893	C	UGT2B11, UGT2B7	4:69113183	5 × 10^−15^		
rs2282679	G	GC	4:71742666	1 × 10^−200^		
rs2205262	C	LINC00536	8:115999659	5 × 10^−11^		
rs7129781	C	CYP2R1	11:14890871	4 × 10^−33^		
rs4944958	A	NADSYN1	11:71457027	9 × 10^−143^		
rs964184	G	ZPR1	11:116778201	3 × 10^−14^		
rs10859995	T	HAL	12:95981904	3 × 10^−33^		
rs1800588	T	LIPC, ALDH1A2	15:58431476	2 × 10^−10^		
rs55791371	C	SMARCA4	19:11077477	1 × 10^−9^		
rs10426201	G	SULT2A1	19:47881492	8 × 10^−20^		
rs17217119	G	BCAS1, CYP24A1	20:54126051	5 × 10^−16^		
rs11723621	G	GC	4:71749645	2 × 10^−24^	GCST90101732	34852423
rs7041	G	GC	4:71752617	2 × 10^−9^		
rs11023332	G	PDE3B	11:14762564	3 × 10^−11^		
rs12785878	G	NADSYN1	11:71456403	2 × 10^−27^	GCST000697	20541252
rs10741657	G	CALCB, CYP2R1	11:14893332	3 × 10^−20^		

**Table 2:** Enrichment analysis of the genes associated with a VD deficiency obtained in disgenet2r (R package). The ratio column represents the number of genes from the input list that are associated with the disease. The BgRatio are the number of genes associated with the disease from the length of the universe, in the example, all genes in DisGeNET (the curated or background genes). The *p*-values resulting from the multiple Fisher tests are corrected for the false discovery rate (FDR) using the Benjamini–Hochberg (BH) method.

Description	FDR	Ratio	BgRatio
Vitamin D3 measurement	5.029 × 10^−11^	5/14	14/21,666
Vitamin D measurement	5.029 × 10^−11^	5/14	14/21,666
Rickets	2.933 × 10^−5^	4/14	72/21,666
Metabolic Syndrome X	2.933 × 10^−5^	8/14	1125/21,666
Serum albumin measurement	3.233 × 10^−5^	6/14	433/21,666
Glucose measurement	3.689 × 10^−5^	4/14	89/21,666
Elevated blood glucose level	3.689 × 10^−5^	4/14	89/21,666
High density lipoprotein cholesterol level quantitative trait locus 12	4.847 × 10^−5^	2/14	2/21,666
Coronary heart disease	1.732 × 10^−4^	8/14	1576/21,666
Vitamin D Deficiency	2.267 × 10^−4^	4/14	153/21,666

**Table 3:** Enrichment analysis of the genes associated with COVID-19, obtained in disgenet2r (R package)**.** The ratio column represents the number of genes from the input list that are associated with the disease. The BgRatio are the number of genes associated with the disease from the length of the universe, in the example, all genes in DisGeNET (the curated or background genes). The *p*-values resulting from the multiple Fisher tests are corrected for the false discovery rate (FDR) using the Benjamini–Hochberg (BH) method.

Description	FDR	Ratio	BgRatio
Primary biliary cirrhosis	3.305 × 10^−4^	8/29	478/21,666
Coughing	4.286 × 10^−4^	6/29	235/21,666
Reticulocyte count (procedure)	4.286 × 10^−4^	6/29	234/21,666
Eosinophilia-Myalgia Syndrome, L-Tryptophan-Related	9.429 × 10^−4^	2/29	2/21,666
Liver diseases	1.163 × 10^−3^	9/29	1019/21,666
Rales	1.163 × 10^−3^	3/29	23/21,666
Sarcoidosis, Pulmonary	1.163 × 10^−3^	4/29	81/21,666
Inclusion Body Myositis (disorder)	1.163 × 10^−3^	4/29	87/21,666
Juvenile Graves disease	1.163 × 10^−3^	2/29	3/21,666
Podoconiosis	1.163 × 10^−3^	2/29	3/21,666

## Data Availability

Links to resources and tools used in this study have been provided in the text. Datasets (list of genes) used are provided in the supplementary material.

## Supplementary Materials

The following supporting information can be downloaded at: https://www.mdpi.com/article/10.3390/ijms232214256/s1. Figure S1: Linkage disequilibrium for vitamin D deficiency; Figure S2: Linkage disequilibrium for COVID-19 (chromosome 3); Figure S3: Linkage disequilibrium for COVID-19 (chromosome 9, 19 and 21); Figure S4: Expression heat map by genes and tissues (COVID-19); Figure S5: REVIGO for vitamin d deficiency and covid 19; Figure S6: Enrichment analysis of COVID-19 genes regarding GO terms in MSigDB on immunological signatures; Figure S7: Vitamin D deficiency gene-disease network; Figure S8: Vitamin D deficiency gene-disease heat map; Figure S9: COVID-19 gene-disease network; Figure S10: COVID-19 gene-disease heat map; Figure S11: Enrichment analysis of VD deficiency and the VDR gene; Table S1: Genes and variants associated with COVID-19; Table S2: GWAS catalog of vitamin D deficiency; Table S3: GWAS catalog of COVID-19.
