# Supplementary material for: Vitamin D Deficiency and COVID-19: A Biological Database Study on Pathways and Gene-Disease Associations
Source: Int J Mol Sci. 2022 Nov 17;23(22):14256. doi: 10.3390/ijms232214256 (PMC9699081; doi:10.3390/ijms232214256)
Supplement: Supplementary file 1 [file ijms-23-14256-s001.zip › ijms-2045718-supplementary.pdf]

Supplementary material

Figure S1. Linkage disequilibrium for vitamin D deficiency 2

Table S1. Genes and variants associated with COVID-19 3

Figure S2. Linkage disequilibrium for COVID-19 (chromosome 3) 6

Figure S3. Linkage disequilibrium for COVID-19 (chromosome 9, 19 and 21) 7

Table S2. GWAS catalog of vitamin D deficiency 8

Table S3. GWAS catalog of COVID-19 12

Figure S4. Expression heat map by genes and tissues (COVID-19) 25

Figure S5. REVIGO for vitamin d deficiency and covid 19 26

Figure S6. Enrichment analysis of COVID-19 genes regarding GO terms in MSigDB on immunological signatures. 27

Figure S7. Vitamin D deficiency gene-disease network 29

Figure S8. Vitamin D deficiency gene-disease heat map 30

Figure S9. COVID-19 gene-disease network 31

Figure S10. COVID-19 gene-disease heat map 32

Figure S11. Enrichment analysis of VD deficiency and the VDR gene 33

Figure S1. Linkage disequilibrium for vitamin D deficiency

A

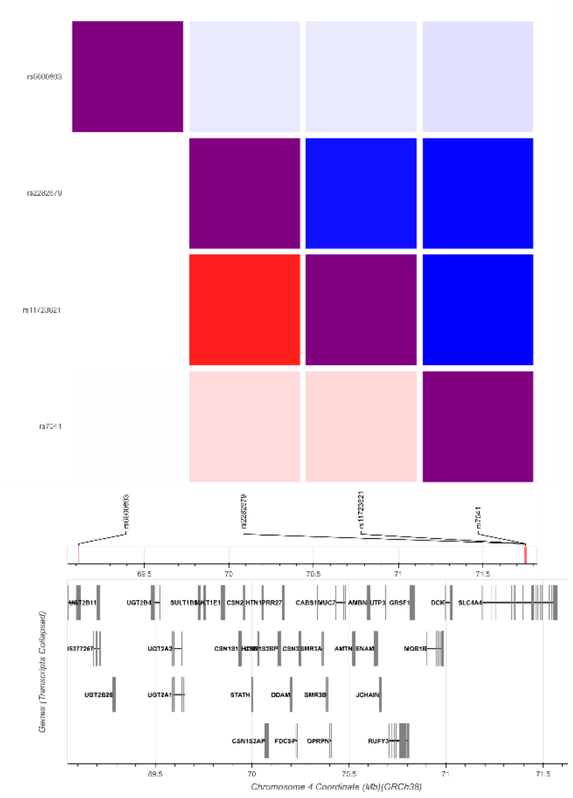

B

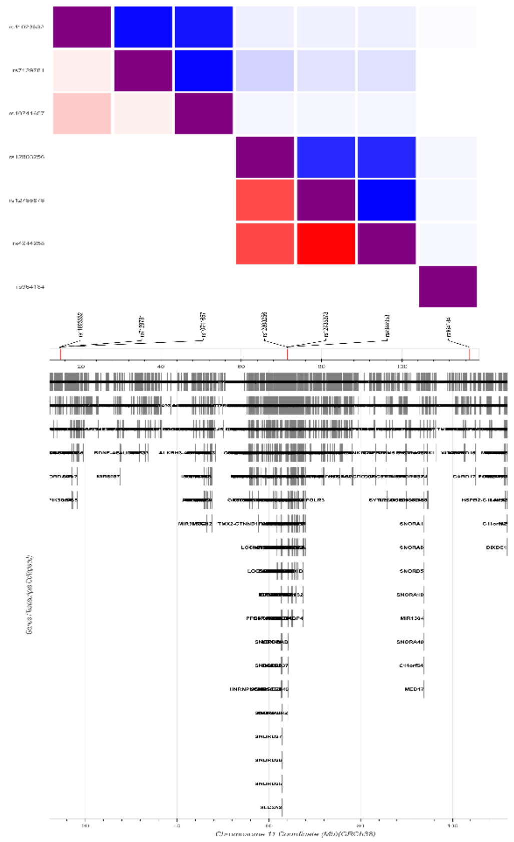

Figure S1. Linkage disequilibrium on chromosome 4 (A) and chromosome 11 (B) for vitamin D deficiency. The degree of correlation between variants within the same chromosome is shown (the higher the degree of correlation, the more likely it is). that these variants are inherited together). Variants with linkage disequilibrium are represented in red. Source: LDLINK

Table S1. Genes and variants associated with COVID-19

Variant	Risk allele	Gene	Location (chr: position)	P-value	Study accession
rs657152	A	ABO	9:133263862	5×10^{-8}	GCST90000255
rs71325088	C	LZTFL1, SLC6A20	3:45821460	3×10^{-54}	GCST010936
rs143334143	A	CCHCR1	6:31153649	2×10^{-10}	
rs6489867	T	OAS1	12:112925745	1×10^{-9}	
rs11085727	T	TYK2	19:10355447	1×10^{-13}	
rs114301457	T	EFNA4	1:155066988	7×10^{-10}	GCST90104030
rs41264915	A	THBS3-AS1, THBS3	1:155197995	1×10^{-12}	
rs73064425	T	LZTFL1	3:45859597	2×10^{-133}	
rs343320	A	PLSCR1	3:146517122	5×10^{-9}	
rs56162149	T	ACSL6	5:131995059	8×10^{-11}	
rs9271609	T	HLA-DRB1, HLA-DQA1	6:32623820	3×10^{-9}	
rs28368148	G	IFNA10	9:21206606	2×10^{-9}	
rs61882275	G	ELF5	11:34482745	2×10^{-10}	
rs56106917	G	GALNT9, FBRSL1	12:132489231	2×10^{-9}	
rs9577175	T	ATP11A, MCF2L	13:112889041	4×10^{-11}	
rs4424872	T	RGMA	15:93046840	9×10^{-13}	
rs117169628	A	SLC22A31	16:89196249	4×10^{-9}	
rs2532300	T	KANSL1	17:46152620	4×10^{-9}	
rs3848456	A	RNU6-1313P, TAC4	17:49863260	4×10^{-11}	
rs12610495	G	DPP9	19:4717660	4×10^{-36}	
rs73510898	A	ZGLP1	19:10305768	2×10^{-11}	
rs34536443	C	TYK2	19:10352442	7×10^{-17}	
rs368565	T	FUT2	19:48697960	4×10^{-11}	
rs17860115	A	IFNAR2	21:33230000	1×10^{-21}	
rs8178521	T	IL10RB	21:33287378	4×10^{-12}	
rs35370143	TAC	LINC00649	21:33959663	1×10^{-9}	
rs60870724	C	CEP97, NXPE3	3:101776487	7×10^{-14}	GCST90103486

rs8176719	TC	ABO	9:133257522	4×10^{-19}	
rs73062389	A	SLC6A20	3:45793925	4×10^{-19}	GCST90103487
rs879055593	T	ABO	9:133271182	7×10^{-34}	GCST90096800
rs190509934	C	ACE2	X:15602217	5×10^{-13}	
rs2531743	G	LZTFL1, SLC6A20	3:45796808	3×10^{-12}	GCST90103428
rs754119466	G	-	Mapping not available	3×10^{-9}	GCST90093366
rs146100088	¿?	MYRIP	3:39838227	7×10^{-8}	GCST90027246
rs35044562	¿?	LZTFL1	3:45867532	4×10^{-14}	
rs183817891	¿?	XCR1	3:46053730	1×10^{-11}	
rs138476573	¿?	PAFAH1B2P2, RMST	12:97636026	8×10^{-8}	
rs11385942	¿?	-	Mapping not available	3×10^{-16}	GCST012399
rs10774671	A	OAS1	12:112919388	4×10^{-13}	GCST90095123
rs77534576	T	RNU6-1313P, TAC4	17:49863303	4×10^{-9}	
rs2109069	A	DPP9	19:4719431	1×10^{-21}	
rs74956615	A	RAVER1	19:10317045	1×10^{-11}	
rs10490770	C	SLC6A20, LZTFL1	3:45823240	1×10^{-73}	GCST90095124
rs1886814	C	FOXP4-AS1	6:41534945	1×10^{-9}	
rs72711165	C	TMEM65	8:124324323	2×10^{-9}	
rs1819040	A	KANSL1	17:46142465	2×10^{-10}	
rs13050728	C	IFNAR2	21:33242905	3×10^{-20}	
rs11919389	C	PDCL3P4	3:101705614	3×10^{-15}	GCST90095125
rs2271616	T	SLC6A20	3:45796521	2×10^{-34}	
rs912805253	T	ABO	9:133274084	1×10^{-39}	
rs9411378	C	ABO	9:133270015	5×10^{-20}	GCST90027245
rs6603626	¿?	LAMP2, RNU7-37P	X:120484021	6×10^{-8}	
rs13078854	G	SLC6A20, LZTFL1	3:45820440	2×10^{-18}	GCST90027249
rs140221038	¿?	FYCO1	3:45954233	1×10^{-13}	
rs111793438	¿?	XCR1	3:46081695	2×10^{-11}	
rs1173773	C	NPR3	5:32750877	5×10^{-8}	GCST012002
rs62575331	G	U2	9:115647521	6×10^{-8}	GCST012001

Table S1. Genes and variants associated with COVID-19. Information on the genes to which these variants belong to, the risk allele of each SNP, location in the genome (chromosome: chr) for hg38 coordinates, and P-Value (existing degree of significance between that variant and the disease). Study accession number obtained from GWAS catalog.

Figure S2. Linkage disequilibrium for COVID-19 (chromosome 3)

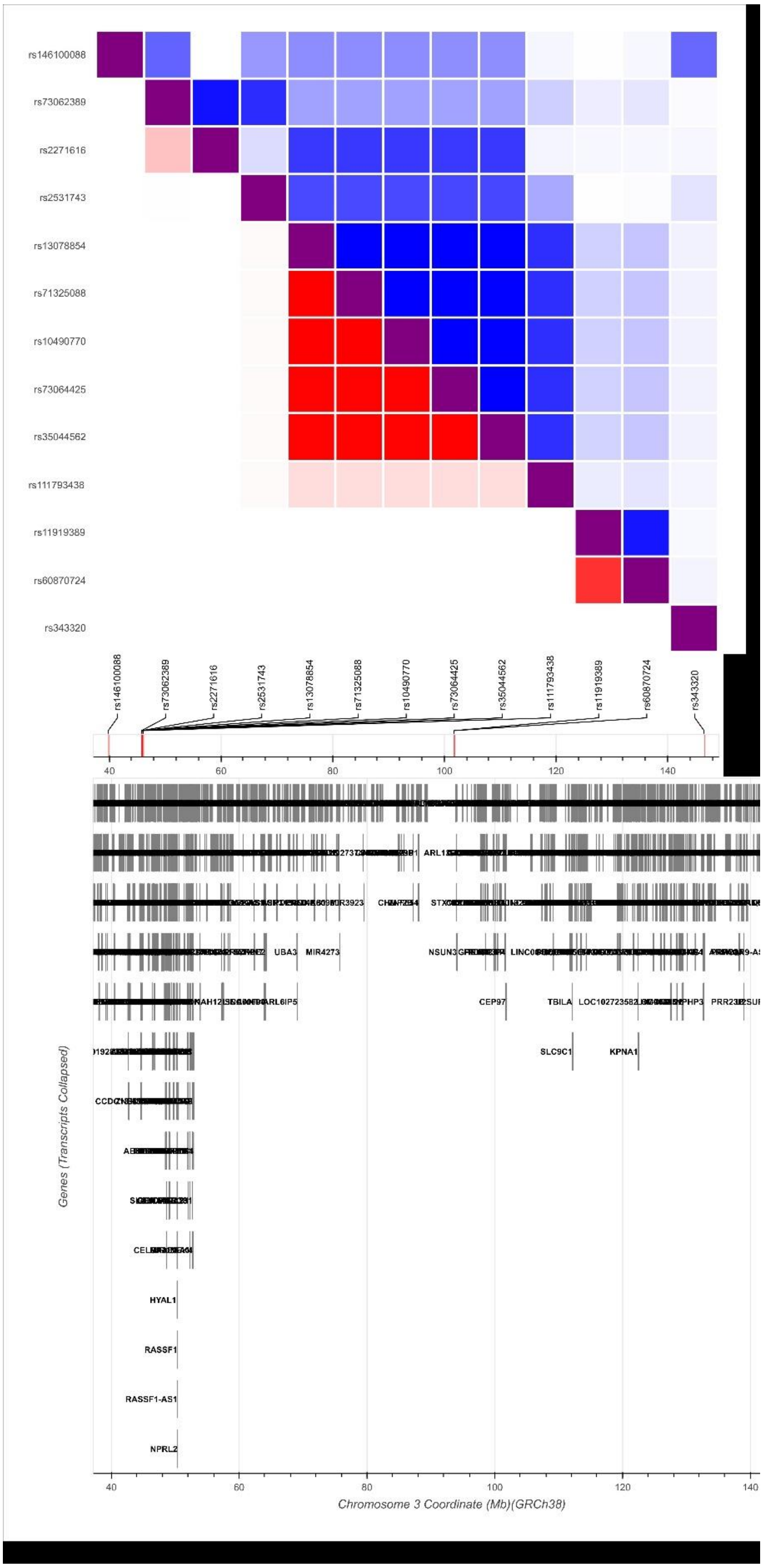

Figure S2. Linkage disequilibrium on chromosome 3 for COVID-19. The degree of correlation between variants within the same chromosome is shown (the higher the degree of correlation, the more likely it is). that these variants are inherited together). Variants with linkage disequilibrium are represented in red. Source: LDLINK

Figure S3. Linkage disequilibrium for COVID-19 (chromosome 9, 19 and 21)

A

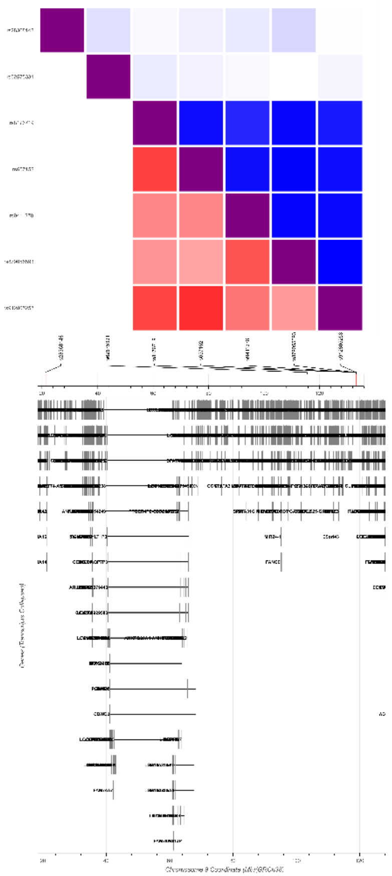

B

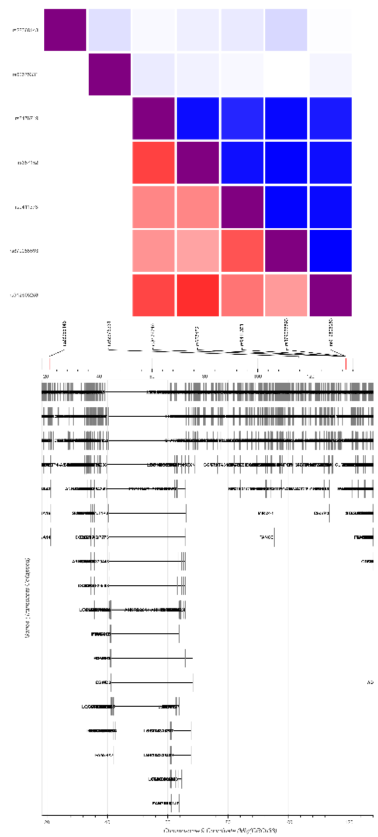

C

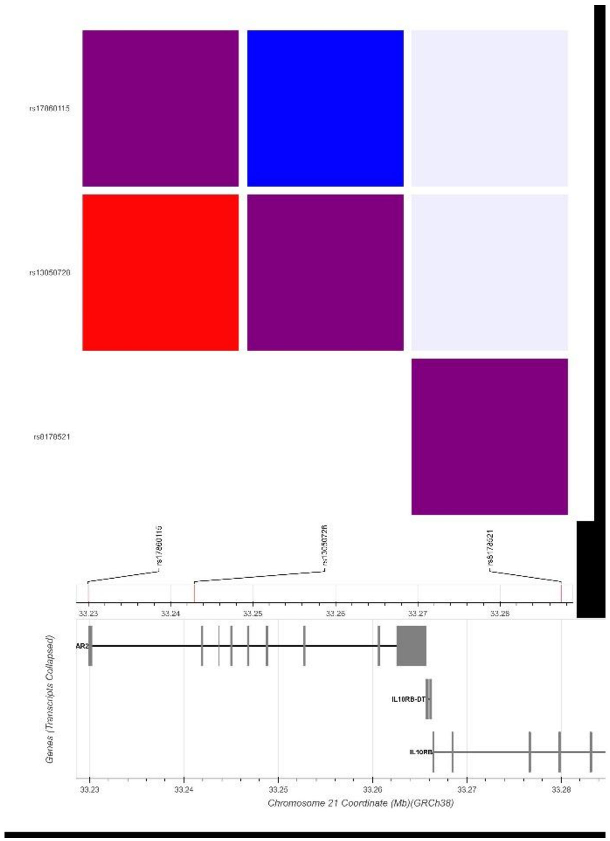

Figure S3. Linkage disequilibrium on chromosome 9 (A), chromosome 19 (B) and chromosome 21 (C) for COVID-19. The degree of correlation between variants within the same chromosome is shown (the higher the degree of correlation, the more likely it is). that these variants are inherited together). Variants with linkage disequilibrium are represented in red. Source: LDLINK

Table S2. GWAS catalog of vitamin D deficiency

Variant and risk allele	P-value	RAF	Beta	CI	Mapped gene	Reported trait	Trait(s)	Study accession	Location
rs7129781-**C**	4 x 10^{-33}	0.071	0.189 unit increase	[0.16-0.22]	CYP2R1	Vitamin D insufficiency	vitamin D deficiency	GCST90020244	11:14890871
rs4944958-**A**	9 x 10^{-143}	0.203	0.259 unit increase	[0.24-0.28]	NADSYN1	Vitamin D insufficiency	vitamin D deficiency	GCST90020244	11:71457027
rs964184-**G**	3 x 10^{-14}	0.129	0.095 unit increase	[0.071-0.119]	ZPR1	Vitamin D insufficiency	vitamin D deficiency	GCST90020244	11:116778201
rs10859995-**T**	3 x 10^{-33}	0.42	0.105 unit decrease	[0.087-0.123]	HAL	Vitamin D insufficiency	vitamin D deficiency	GCST90020244	12:95981904
rs1532085-**A**	1 x 10^{-8}	0.383	0.05 unit increase	[0.032-0.068]	ALDH1A2	Vitamin D insufficiency	vitamin D deficiency	GCST90020244	15:58391167
rs1800588-**T**	2 x 10^{-10}	0.212	0.066 unit increase	[0.046-0.086]	LIPC, ALDH1A2	Vitamin D insufficiency	vitamin D deficiency	GCST90020244	15:58431476
rs55791371-**C**	1 x 10^{-9}	0.121	0.081 unit decrease	[0.056-0.106]	SMARCA4	Vitamin D insufficiency	vitamin D deficiency	GCST90020244	19:11077477
rs10426201-**G**	8 x 10^{-20}	0.169	0.106 unit decrease	[0.082-0.13]	SULT2A1	Vitamin D insufficiency	vitamin D deficiency	GCST90020244	19:47881492
rs3750297-**A**	3 x 10^{-10}	0.283	0.059 unit increase	[0.041-0.077]	PADI1	Vitamin D insufficiency	vitamin D deficiency	GCST90020244	1:17233181

rs12123821-**T**	6×10^{-26}	0.047	0.227 unit decrease	[0.18-0.27]	FLG-AS1	Vitamin D insufficiency	vitamin D deficiency	GCST90020244	1:152206676
rs4845491-**C**	7×10^{-10}	0.054	0.119 unit decrease	[0.082-0.156]	SMCP, LCE6A	Vitamin D insufficiency	vitamin D deficiency	GCST90020244	1:152877093
rs8123293-**G**	3×10^{-8}	0.113	0.076 unit decrease	[0.049-0.103]	BCAS1, CYP24A1	Vitamin D insufficiency	vitamin D deficiency	GCST90020244	20:54102016
rs17217119-**G**	5×10^{-16}	0.193	0.086 unit increase	[0.064-0.108]	BCAS1, CYP24A1	Vitamin D insufficiency	vitamin D deficiency	GCST90020244	20:54126051
rs3755322-**G**	6×10^{-12}	0.083	0.104 unit increase	[0.075-0.133]	UGT1A9, UGT1A5, UGT1A10, UGT1A7, UGT1A8, UGT1A6	Vitamin D insufficiency	vitamin D deficiency	GCST90020244	2:233713141
rs6600893-**C**	5×10^{-15}	0.452	0.067 unit increase	[0.049-0.085]	UGT2B11, UGT2B7	Vitamin D insufficiency	vitamin D deficiency	GCST90020244	4:69113183
rs2282679-**G**	1×10^{-200}	0.274	0.429 unit increase	[0.41-0.45]	GC	Vitamin D insufficiency	vitamin D deficiency	GCST90020244	4:71742666
rs2205262-**C**	5×10^{-11}	0.429	0.057 unit decrease	[0.039-0.075]	LINC00536	Vitamin D insufficiency	vitamin D deficiency	GCST90020244	8:115999659
rs11723621-?	2×10^{-24}	NR	1.28 ng/mL increase	[-18.7708-21.3308]	GC	Vitamin D insufficiency	vitamin D deficiency	GCST90101732	4:71749645

rs11023332-?	3 x 10^{-11}	NR	0.77 ng/mL increase	[-12.2444-13.7844]	PDE3B	Vitamin D insufficiency	vitamin D deficiency	GCST90101732	11:14762564
rs7041-?	2 x 10^{-9}	NR	0.79 ng/mL increase	[-11.0288-12.6088]	GC	Vitamin D insufficiency	vitamin D deficiency	GCST90101732	4:71752617
rs12803256-?	4 x 10^{-8}	NR	0.65 ng/mL increase	[-10.13-11.43]	ACTE1P	Vitamin D insufficiency	vitamin D deficiency	GCST90101732	11:71421822
rs3831470-?	1 x 10^{-6}	NR	0.58 ng/mL increase	[-9.024-10.184]	NADSYN1	Vitamin D insufficiency	vitamin D deficiency	GCST90101732	11:71454899
rs78359207-?	2 x 10^{-6}	NR	0.59 ng/mL increase	[-8.7788-9.9588]	NPFFR2	Vitamin D insufficiency	vitamin D deficiency	GCST90101732	4:72032699
rs55715230-?	3 x 10^{-6}	NR	0.66 ng/mL increase	[-8.5324-9.8524]	PACRG	Vitamin D insufficiency	vitamin D deficiency	GCST90101732	6:162756538
rs306141-?	3 x 10^{-6}	NR	0.6 ng/mL increase	[-8.5336-9.7336]	LINC01247, LINC01824	Vitamin D insufficiency	vitamin D deficiency	GCST90101732	2:6458749
rs17382663-?	5 x 10^{-6}	NR	0.91 ng/mL decrease	[-8.008-9.828]	PVT1	Vitamin D insufficiency	vitamin D deficiency	GCST90101732	8:128090786
rs58788626-?	7 x 10^{-6}	NR	0.84 ng/mL decrease	[-7.98-9.66]	PCNX1, MAP3K9-DT	Vitamin D insufficiency	vitamin D deficiency	GCST90101732	14:70865550
rs12785878-?	2 x 10^{-27}	0.23	'-	'-	NADSYN1	Vitamin D insufficiency	vitamin D deficiency	GCST000697	11:71456403
rs2282679-?	2 x 10^{-109}	0.29	'-	'-	GC	Vitamin D insufficiency	vitamin D deficiency	GCST000697	4:71742666

rs10741657 -?	3 x 10^{-20}	0.40	'-	'-	CALCB, CYP2R1	Vitamin D insufficiency	vitamin D deficiency	GCST000697	11:14893332

Table S2. GWAS catalog of vitamin D deficiency**.** Complete information for sets of genetic variants, regarding relative allele frequencies (RAF) and effect sizes of the associations.

Table S3. GWAS catalog of COVID-19

Variant and risk allele	P-value	RAF	Beta	CI	Mapped gene	Reported trait	Trait(s)	Study accession	Location
rs657152-**A**	5 x 10-8	0.35	'-	[1.20-1.47]	ABO	Severe COVID-19 infection with respiratory failure (analysis I)	COVID-19	GCST90000255	9:133263862
rs11385942-**GA**	9 x 10-12	0.09	'-	[1.70-2.61]	'-	Severe COVID-19 infection with respiratory failure (analysis II)	COVID-19	GCST90000256	Mapping not available
rs71325088-**C**	3 x 10-54	NR	'-	[1.73-2.0]	LZTFL1, SLC6A20	COVID-19 (critical illness vs population)	COVID-19	GCST010936	3:45821460
rs143334143-**A**	2 x 10-10	NR	'-	[1.27-1.48]	CCHCR1	COVID-19 (critical illness vs population)	COVID-19	GCST010936	6:31153649
rs6489867-**T**	1 x 10-9	NR	'-	[1.14-1.25]	OAS1	COVID-19 (critical illness vs population)	COVID-19	GCST010936	12:112925745
rs11085727-**T**	1 x 10-13	NR	'-	[1.18-1.31]	TYK2	COVID-19 (critical	COVID-19	GCST010936	19:10355447

						illness vs population)			
rs13050728-**T**	5 x 10-12	NR	'-	[1.16-1.28]	IFNAR2	COVID-19 (critical illness vs population)	COVID-19	GCST010936	21:33242905
rs1123573-**A**	2 x 10-8	NR	'-	'-	BCL11A	COVID-19 (critical illness vs population or mild symptoms)	COVID-19	GCST90104034	2:60480453
rs2496644-**A**	2 x 10-8	NR	'-	'-	FOXP4-AS1, LINC01276	COVID-19 (critical illness vs population or mild symptoms)	COVID-19	GCST90104034	6:41515007
rs13071258-?	2 x 10-8	NR	'-	[2.98-3.75]	'-	COVID-19 (critical illness vs population or mild symptoms)	COVID-19	GCST90104036	Mapping not available
rs114301457-**T**	7 x 10-10	0.0058	'-	[1.82-3.16]	EFNA4	COVID-19 (critical illness vs population or mild symptoms)	COVID-19	GCST90104030	1:155066988

rs41264915-**A**	1 x 10-12	0.89	'-	[1.19-1.37]	THBS3-AS1, THBS3	COVID-19 (critical illness vs population or mild symptoms)	COVID-19	GCST90104030	1:155197995
rs73064425-**T**	2 x 10-133	0.077	'-	[2.51-2.94]	LZTFL1	COVID-19 (critical illness vs population or mild symptoms)	COVID-19	GCST90104030	3:45859597
rs343320-**A**	5 x 10-9	0.081	'-	[1.16-1.35]	PLSCR1	COVID-19 (critical illness vs population or mild symptoms)	COVID-19	GCST90104030	3:146517122
rs56162149-**T**	8 x 10-11	0.17	'-	[1.13-1.26]	ACSL6	COVID-19 (critical illness vs population or mild symptoms)	COVID-19	GCST90104030	5:131995059
rs9271609-**T**	3 x 10-9	0.65	'-	[1.09-1.19]	HLA-DRB1, HLA-DQA1	COVID-19 (critical illness vs population or mild symptoms)	COVID-19	GCST90104030	6:32623820

rs28368148-**G**	2 x 10-9	0.013	'-	[1.45-2.09]	IFNA10	COVID-19 (critical illness vs population or mild symptoms)	COVID-19	GCST90104030	9:21206606
rs61882275-**G**	2 x 10-10	0.62	'-	[1.1-1.2]	ELF5	COVID-19 (critical illness vs population or mild symptoms)	COVID-19	GCST90104030	11:34482745
rs56106917-**G**	2 x 10-9	0.49	'-	[1.09-1.18]	GALNT9, FBRSL1	COVID-19 (critical illness vs population or mild symptoms)	COVID-19	GCST90104030	12:132489231
rs9577175-**T**	4 x 10-11	0.23	'-	[1.12-1.24]	ATP11A, MCF2L	COVID-19 (critical illness vs population or mild symptoms)	COVID-19	GCST90104030	13:112889041
rs4424872-**T**	9 x 10-13	0.0079	'-	[1.87-3.01]	RGMA	COVID-19 (critical illness vs population or mild symptoms)	COVID-19	GCST90104030	15:93046840

rs117169628-**A**	4 x 10-9	0.15	'-	[1.12-1.26]	SLC22A31	COVID-19 (critical illness vs population or mild symptoms)	COVID-19	GCST90104030	16:89196249
rs2532300-**T**	4 x 10-9	0.77	'-	[1.1-1.22]	KANSL1	COVID-19 (critical illness vs population or mild symptoms)	COVID-19	GCST90104030	17:46152620
rs3848456-**A**	4 x 10-11	0.029	'-	[1.33-1.7]	RNU6-1313P, TAC4	COVID-19 (critical illness vs population or mild symptoms)	COVID-19	GCST90104030	17:49863260
rs12610495-**G**	4 x 10-36	0.31	'-	[1.27-1.38]	DPP9	COVID-19 (critical illness vs population or mild symptoms)	COVID-19	GCST90104030	19:4717660
rs73510898-**A**	2 x 10-11	0.093	'-	[1.19-1.37]	ZGLP1	COVID-19 (critical illness vs population or mild symptoms)	COVID-19	GCST90104030	19:10305768

rs34536443-**C**	7 x 10-17	0.05	'-	[1.36-1.65]	TYK2	COVID-19 (critical illness vs population or mild symptoms)	COVID-19	GCST90104030	19:10352442
rs368565-**T**	4 x 10-11	0.44	'-	[1.1-1.2]	FUT2	COVID-19 (critical illness vs population or mild symptoms)	COVID-19	GCST90104030	19:48697960
rs17860115-**A**	1 x 10-21	0.32	'-	[1.19-1.3]	IFNAR2	COVID-19 (critical illness vs population or mild symptoms)	COVID-19	GCST90104030	21:33230000
rs8178521-**T**	4 x 10-12	0.27	'-	[1.12-1.23]	IL10RB	COVID-19 (critical illness vs population or mild symptoms)	COVID-19	GCST90104030	21:33287378
rs35370143-**TAC**	1 x 10-9	0.083	'-	[1.17-1.36]	LINC00649	COVID-19 (critical illness vs population or mild symptoms)	COVID-19	GCST90104030	21:33959663

rs7528026-**A**	2 x 10-8	NR	'-	'-	TRIM46	COVID-19 (critical illness vs population or mild symptoms)	COVID-19	GCST90104034	1:155175305
rs8176719-**TC**	4 x 10-19	0.337	'-	[1.08-1.13]	ABO	COVID-19	COVID-19	GCST90103486	9:133257522
rs60870724-**C**	7 x 10-14	0.333	'-	[0.88-0.93]	CEP97, NXPE3	COVID-19	COVID-19	GCST90103486	3:101776487
rs73062389-**A**	4 x 10-19	0.064	'-	[1.19-1.31]	SLC6A20	COVID-19	COVID-19	GCST90103487	3:45793925
rs73910904-**A**	4 x 10-8	0.016	'-	[1.41-2.06]	SILC1, LINC01247	COVID-19	COVID-19	GCST90103489	2:6013310
rs190509934-**C**	5 x 10-13	NR	'-	[0.52-0.69]	ACE2	COVID-19 (covid vs negative or unknown)	COVID-19	GCST90096800	X:15602217
rs879055593-**T**	7 x 10-34	NR	'-	[1.081-1.114]	ABO	COVID-19 (covid vs negative or unknown)	COVID-19	GCST90096800	9:133271182
rs2531743-**G**	3 x 10-12	NR	'-	[0.927-0.958]	LZTFL1, SLC6A20	COVID-19 (covid vs negative)	COVID-19	GCST90103428	3:45796808
rs769102632-**A**	3 x 10-8	2.0E-5	'-	[8.37-85.38]	'-	COVID-19	COVID-19	GCST90093364	Mapping not available
rs1256764500-**G**	4 x 10-8	1.0E-5	'-	[8.25-85.77]	'-	COVID-19	COVID-19	GCST90093364	Mapping not available
rs754119466-**G**	3 x 10-9	4.0E-5	'-	[13.61-177.85]	'-	COVID-19 (hospitalize	COVID-19	GCST90093366	Mapping not available

						d vs population)			
rs761377603-**T**	2 x 10-8	3.0E-5	'-	[10.75-140.9]	TES	COVID-19 (hospitalized vs population)	COVID-19	GCST90093366	7:116250230
rs2287960-**T**	4 x 10-8	2.0E-5	'-	[11.17-165.02]	SHC2	COVID-19 (hospitalized vs population)	COVID-19	GCST90093366	19:422238
chr20:58350370-**A**	2 x 10-9	0.0176	0.828 unit decrease	[0.56-1.1]	'-	General population vs COVID-19 exposed negative controls	COVID-19	GCST90094648	Mapping not available
chr10:14687933-**A**	2 x 10-8	0.1295	0.4831 unit increase	[0.31-0.65]	'-	COVID-19 (hospitalized vs population)	COVID-19	GCST90094646	Mapping not available
rs146100088-?	7 x 10-8	NR	1.455542 unit increase	[0.93-1.98]	MYRIP	COVID-19 (hospitalized covid vs population)	COVID-19	GCST90027246	3:39838227
rs183817891-?	1 x 10-11	NR	0.5624724 unit increase	[0.4-0.73]	XCR1	COVID-19 (hospitalized covid vs population)	COVID-19	GCST90027246	3:46053730
rs11385942-?	3 x 10-16	'-	'-	[1.43-1.80]	'-	COVID-19	COVID-19	GCST012399	Mapping not available

chr11:11407970-**C**	2 x 10-8	0.2258	0.4641 unit increase	[0.3-0.63]	'-	COVID-19 (hospitalized vs not hospitalized)	COVID-19	GCST90101882	Mapping not available
rs10774671-**A**	4 x 10-13	0.6518	'-	[1.1422351034880305-1.2604553442434507]	OAS1	COVID-19 (critical illness vs population)	COVID-19	GCST90095123	12:112919388
rs77534576-**T**	4 x 10-9	0.03275	'-	[1.2784125078885984-1.635422073558583]	RNU6-1313P, TAC4	COVID-19 (critical illness vs population)	COVID-19	GCST90095123	17:49863303
rs2109069-**A**	1 x 10-21	0.3158	'-	[1.2014454336815512-1.3203998930516965]	DPP9	COVID-19 (critical illness vs population)	COVID-19	GCST90095123	19:4719431
rs74956615-**A**	1 x 10-11	0.04816	'-	[1.2926896399458667-1.5907330695298332]	RAVER1	COVID-19 (critical illness vs population)	COVID-19	GCST90095123	19:10317045
rs10490770-**C**	1 x 10-73	0.08141	'-	[1.5622524842496601-1.740532881367777]	SLC6A20, LZTFL1	COVID-19 (hospitalized vs population)	COVID-19	GCST90095124	3:45823240
rs1886814-**C**	1 x 10-9	0.04242	'-	[1.1708908156456612-1.35987823139442]	FOXP4-AS1	COVID-19 (hospitalized vs population)	COVID-19	GCST90095124	6:41534945
rs72711165-**C**	2 x 10-9	0.01337	'-	[1.2355706893674907-	TMEM65	COVID-19 (hospitalize	COVID-19	GCST90095124	8:124324323

				1.5180506428830653]		d vs population)			
rs1819040-**A**	2 x 10-10	0.1855	'-	[0.8448494104431585-0.9145683076087429]	KANSL1	COVID-19 (hospitalized vs population)	COVID-19	GCST90095124	17:46142465
rs13050728-**C**	3 x 10-20	0.6511	'-	[0.8338527591429614-0.8886591191741529]	IFNAR2	COVID-19 (hospitalized vs population)	COVID-19	GCST90095124	21:33242905
rs2271616-**T**	2 x 10-34	0.1178	'-	[1.128340128519996-1.1814852813843186]	SLC6A20	SARS-CoV-2 infection	COVID-19	GCST90095125	3:45796521
rs11919389-**C**	3 x 10-15	0.3515	'-	[0.9273018223094552-0.9556267949509863]	PDCL3P4	SARS-CoV-2 infection	COVID-19	GCST90095125	3:101705614
rs912805253-**T**	1 x 10-39	0.6512	'-	[0.8914217954151271-0.9183814681761949]	ABO	SARS-CoV-2 infection	COVID-19	GCST90095125	9:133274084
rs4801778-**T**	1 x 10-8	0.1798	'-	[0.9281405415749607-0.9642334091548289]	PLEKHA4	SARS-CoV-2 infection	COVID-19	GCST90095125	19:48867352
rs142658912-?	3 x 10-8	NR	'-	[2.711-8.048]	GTF2IP23, GTF2IRD1P1	COVID-19 (covid respiratory support vs population)	COVID-19	GCST90027248	7:66879664

rs117479047-?	4 x 10-8	NR	'-	[0.047-0.232]	GNAI2P2, LINC00856	COVID-19 (covid respiratory support vs population)	COVID-19	GCST90027248	10:78069803
rs138476573-?	8 x 10-8	NR	0.7780519 unit increase	[0.5-1.06]	PAFAH1B2P2, RMST	COVID-19 (hospitalized covid vs population)	COVID-19	GCST90027246	12:97636026
rs6603626-?	6 x 10-8	NR	0.0677461 unit increase	[0.043-0.092]	LAMP2, RNU7-37P	COVID-19 (covid vs negative)	COVID-19	GCST90027245	X:120484021
rs35044562-?	4 x 10-14	NR	'-	[1.555-2.114]	LZTFL1	COVID-19 (hospitalized covid vs population)	COVID-19	GCST90027246	3:45867532
rs9411378-**C**	5 x 10-20	NR	'-	[0.829-0.886]	ABO	COVID-19 (covid vs negative)	COVID-19	GCST90027245	9:133270015
rs12564811-?	3 x 10-8	NR	'-	NR	LCE2B, LCE2A	COVID-19 or psoriasis (trans-disease meta-analysis)	psoriasis, COVID-19	GCST011477	1:152693204
rs13078854-**G**	2 x 10-18	NR	'-	[0.527-0.665]	SLC6A20, LZTFL1	COVID-19 (severe respiratory symptoms vs population)	respiratory symptom measurement, COVID-19	GCST90027249	3:45820440

rs140221038-?	1 x 10-13	NR	0.5297379 unit increase	[0.39-0.67]	FYCO1	COVID-19 (severe respiratory symptoms vs population)	respiratory symptom measurement, COVID-19	GCST90027249	3:45954233
rs111793438-?	2 x 10-11	NR	0.379308 unit decrease	[0.27-0.49]	XCR1	COVID-19 (severe respiratory symptoms vs population)	respiratory symptom measurement, COVID-19	GCST90027249	3:46081695
rs1173773-**C**	5 x 10-8	NR	'-	[1.15-1.35]	NPR3	Symptoms in COVID-19 outpatients at risk of severe disease (time to remission)	time to remission of COVID-19 symptoms, COVID-19	GCST012002	5:32750877
rs5913398-?	3 x 10-8	NR	0.5746994 unit increase	[0.37-0.78]	RNU6-995P, HMGN5	COVID-19 (severe respiratory symptoms vs population)	respiratory symptom measurement, COVID-19	GCST90027249	X:81083060
chr22:49597935-**T**	6 x 10-8	0.1909	0.1454 unit increase	[0.093-0.198]	'-	COVID-19 severity score	COVID-19 symptoms measurement, COVID-19	GCST90094650	Mapping not available

rs62575331-**G**	3 x 10-8	0.12	'-	[1.34-1.84]	U2	Symptoms in placebo-treated COVID-19 outpatients at risk of severe disease (time to remission)	time to remission of COVID-19 symptoms, COVID-19, response to placebo	GCST012001	9:115647521

Table S3. GWAS catalog of COVID-19. Complete information for sets of genetic variants, regarding relative allele fre-quencies (RAF) and effect sizes of the associations.

Figure S4. Expression heat map by genes and tissues (COVID-19)

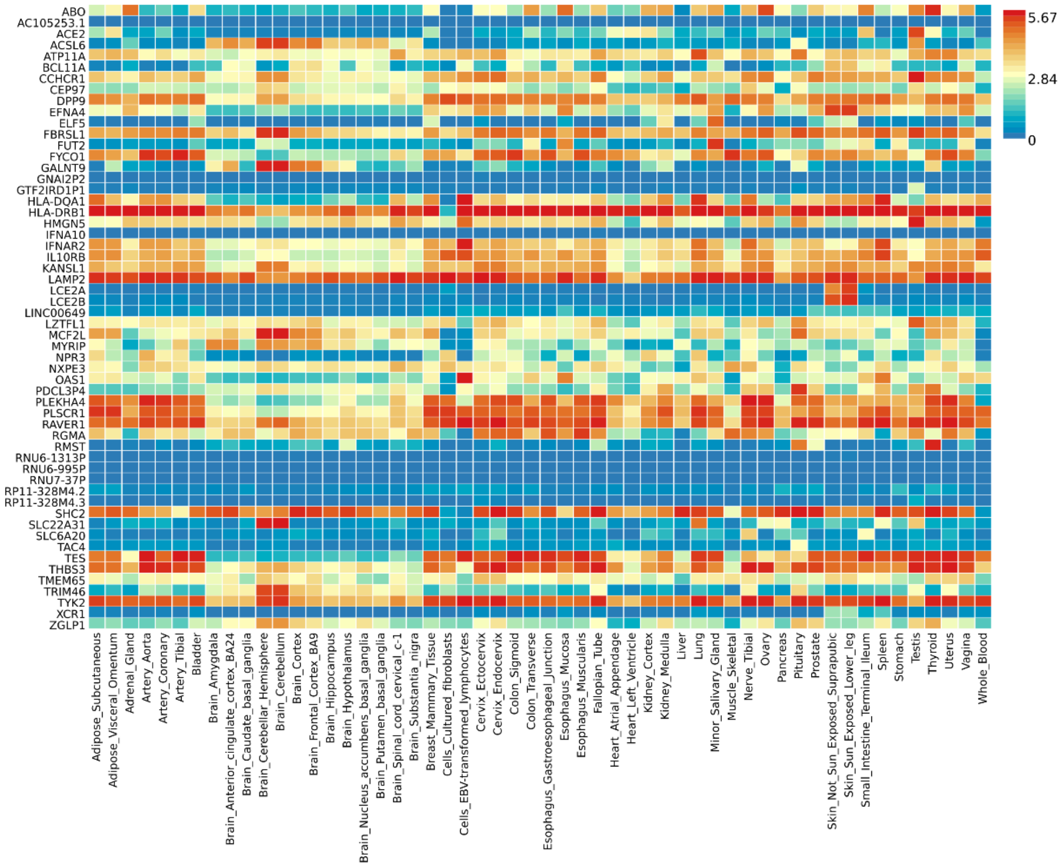

Figure S4. Expression heat map by genes and tissues (COVID-19). The tissue-specific expression patterns are based on GTEx v6 RNA-seq data. Cells in red represent higher expression compared to cells in blue. Gene expression comparisons between tissues (horizontal comparison) within a gene (y axis) are comparable but not those of different genes within a tissue (vertical comparison). Thus, cells in red represent a higher expression of genes in a corresponding tissue compared to other tissues, but do NOT represent higher expression compared to other genes. Source: FUMA

Figure S5. REVIGO for vitamin d deficiency and covid 19

A

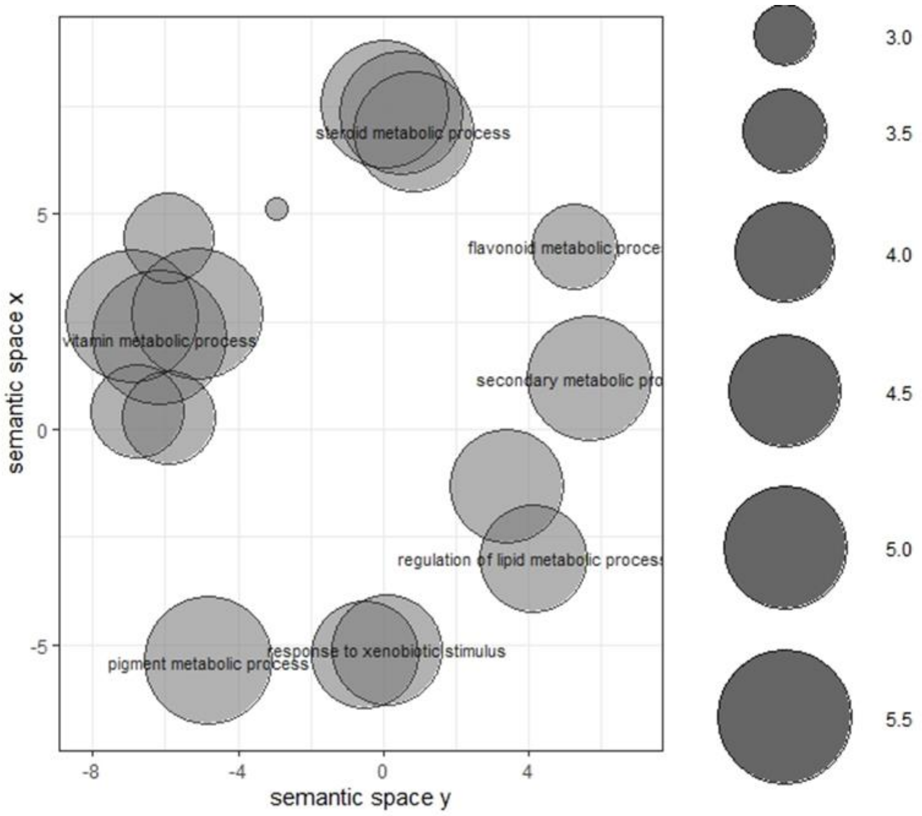

B

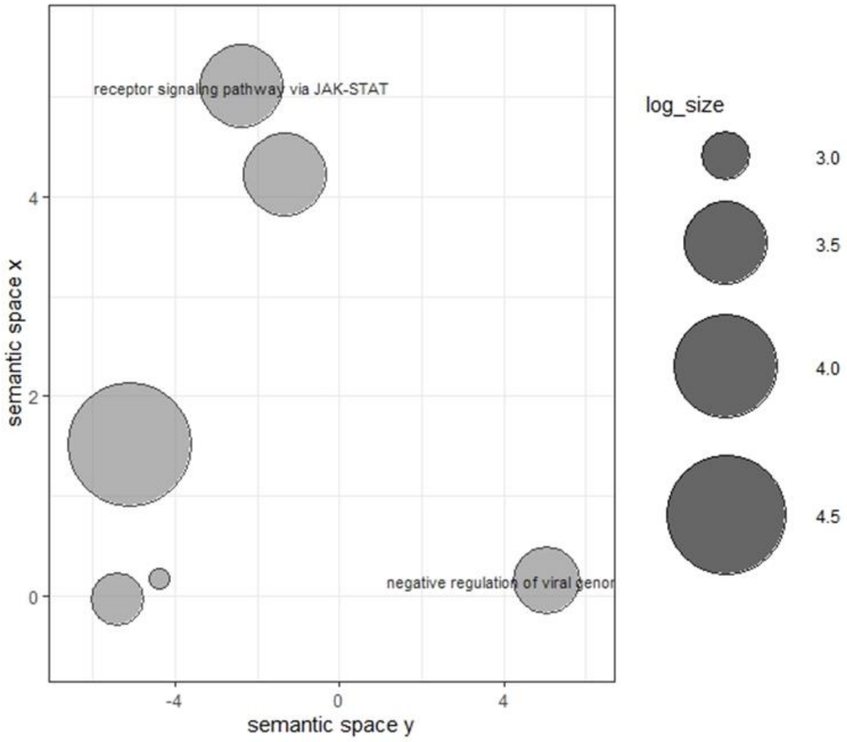

FigureS5: Multidimensional scaling plots obtained in REVIGO platform of the matrix of GO terms (log-scale) for vitamin D deficiency (A) and COVID-19 (B). Semantically similar GO terms are closer in the plot. Source: Revigo

Figure S6. Enrichment analysis of COVID-19 genes regarding GO terms in MSigDB on immunological signatures.

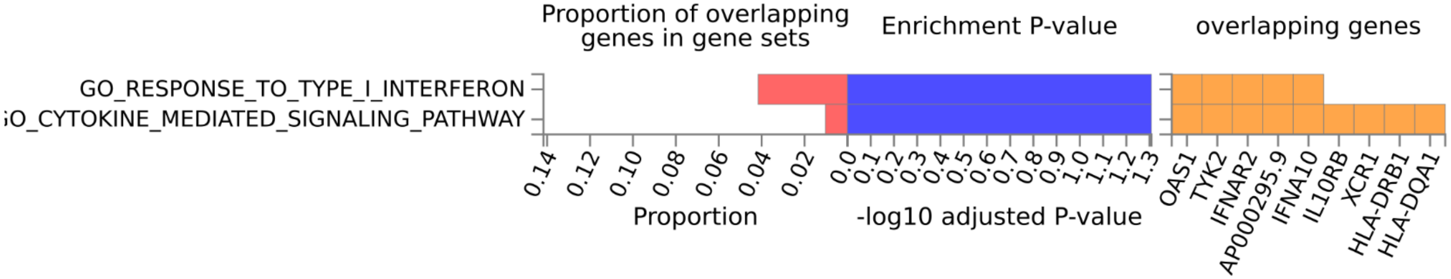

Figure S6: Pathways and processes that are overrepresented in the gene set of interest are shown. Also, only genes present in a given pathway or process are given. Overrepresentation of the gene set of interest (input genes) regarding pathways and functions was tested using the hypergeometric test against pre-defined gene sets (background genes) obtained from MsigDB. Input genes that are overlapping in the pathway or process, the enrichment P-value and the proportion of overlapping genes (input genes relative to the tested gene set) are shown. Source: FUMA

Figure S7. Vitamin D deficiency gene-disease network

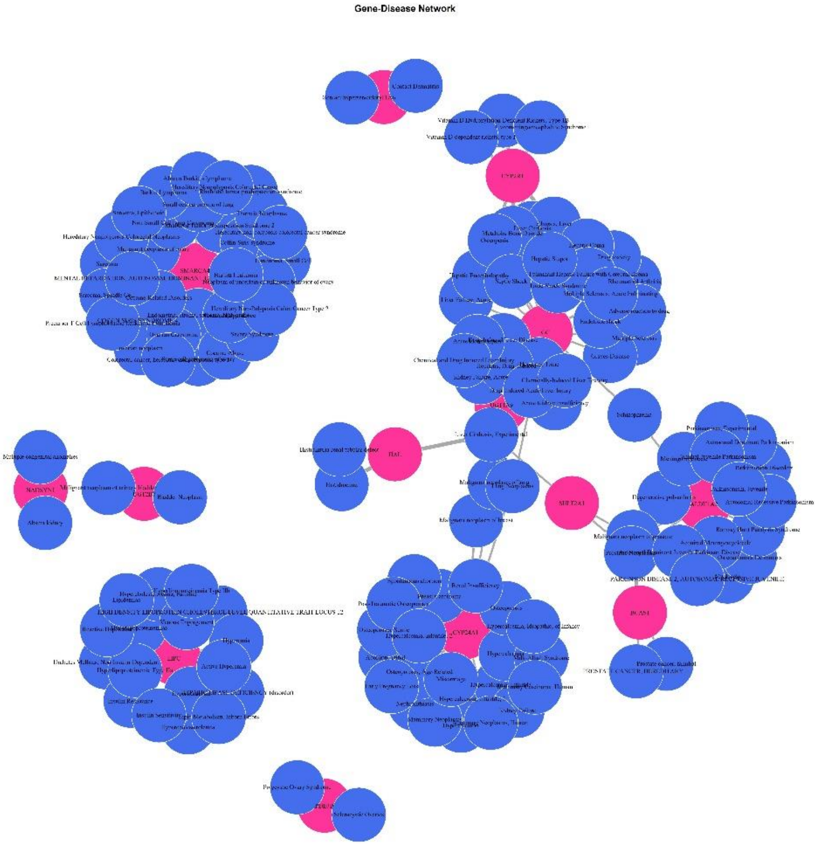

Figure S7. Vitamin D deficiency gene-disease network. In this Network, the pink nodes represent the genes for vitamin D deficiency, the blue nodes for diseases, the lines that join them represent the gene-disease association, and the width of the borders is proportional to the score of the association. Source: disgenet2r (R package).

Figure S8. Vitamin D deficiency gene-disease heat map

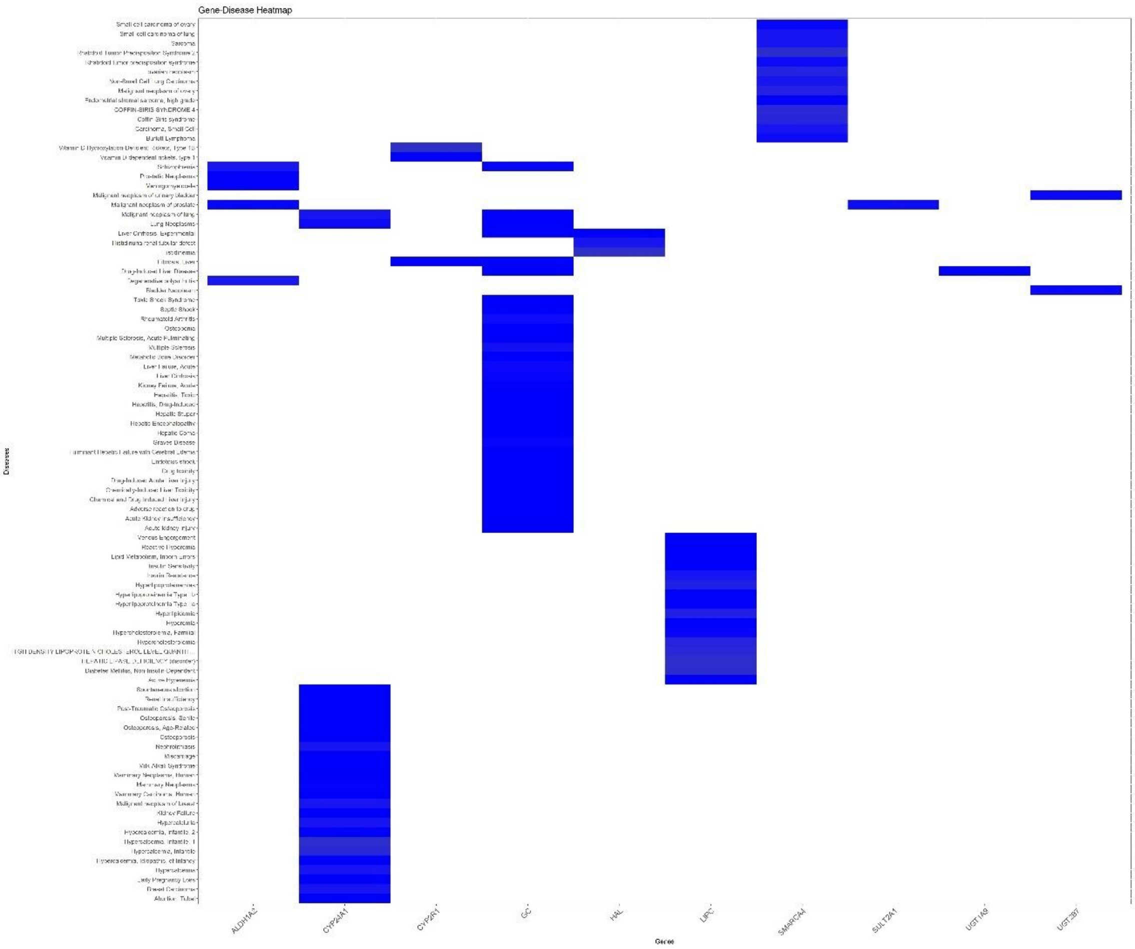

Figure S8. Vitamin D deficiency gene-disease heat map. In this map, the color scale is proportional to the gene-disease association score. The graph shows the 50 GDAs with the highest score. Source: disgenet2r (R package).

Figure S9. COVID-19 gene-disease network

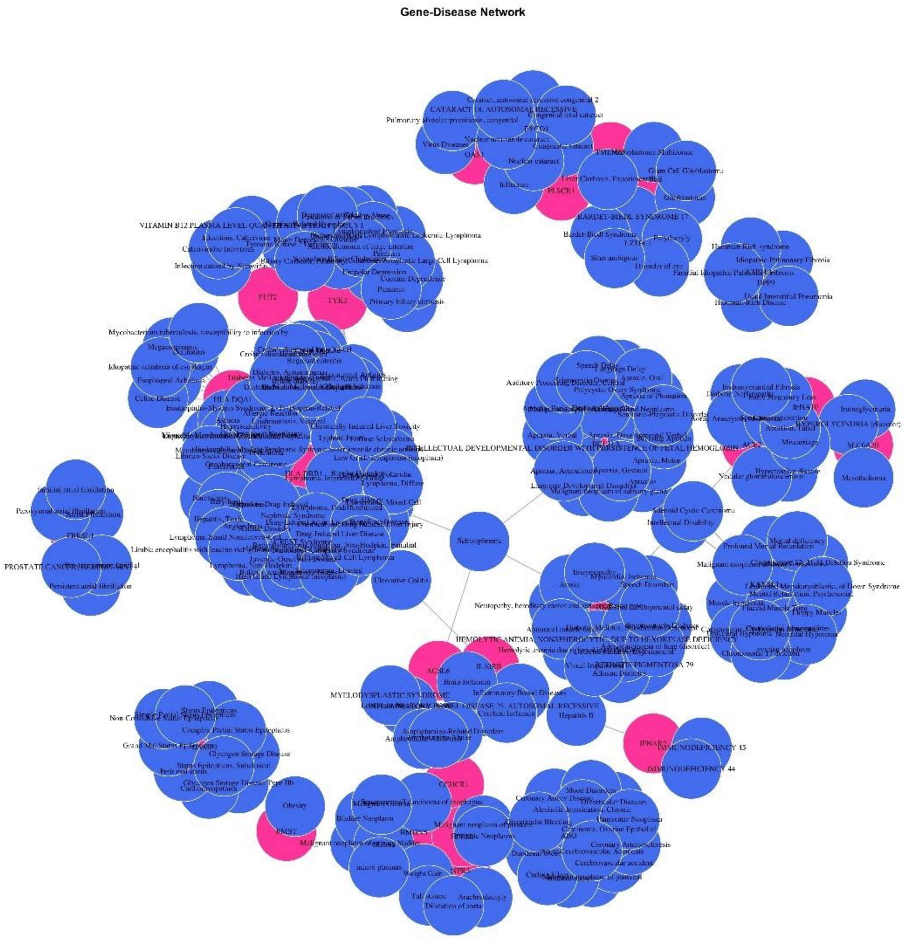

Figure S9. COVID-19 gene-disease network. In this Network, the pink nodes represent the genes for COVID-19, the blue nodes for diseases, the lines that join them represent the gene-disease association, and the width of the borders is proportional to the score of the association. Source: disgenet2r (R package).

Figure S10. COVID-19 gene-disease heat map

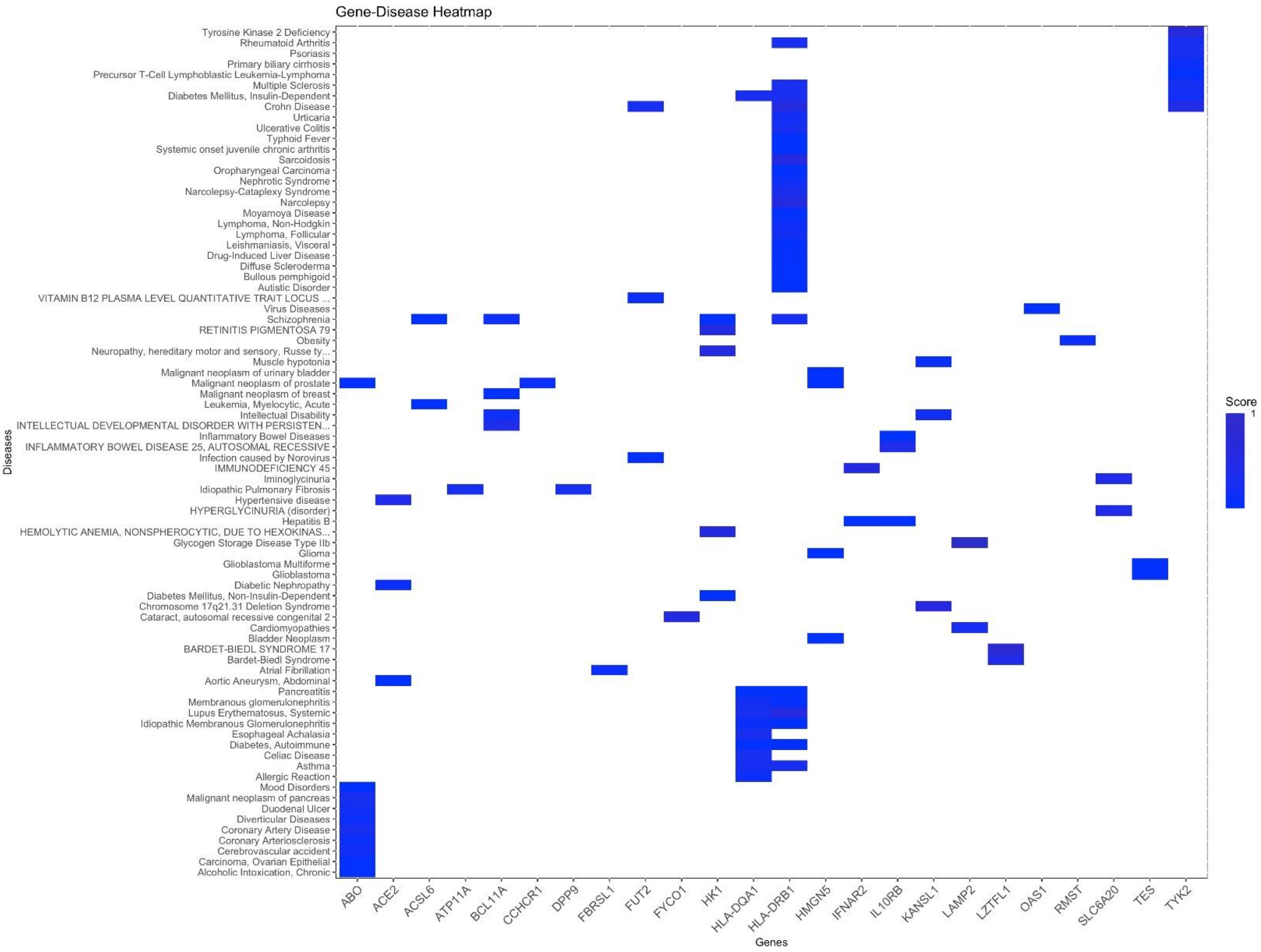

Figure S10. COVID-19 gene-disease heat map. In this map, the color scale is proportional to the gene-disease association score. The graph shows the 50 GDAs with the highest score. Source: disgenet2r (R package).

Figure S11. Enrichment analysis of VD deficiency and the VDR gene

A

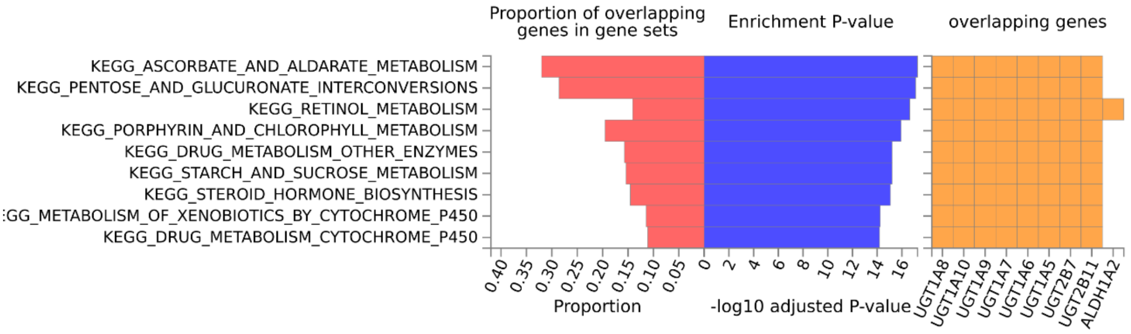

B

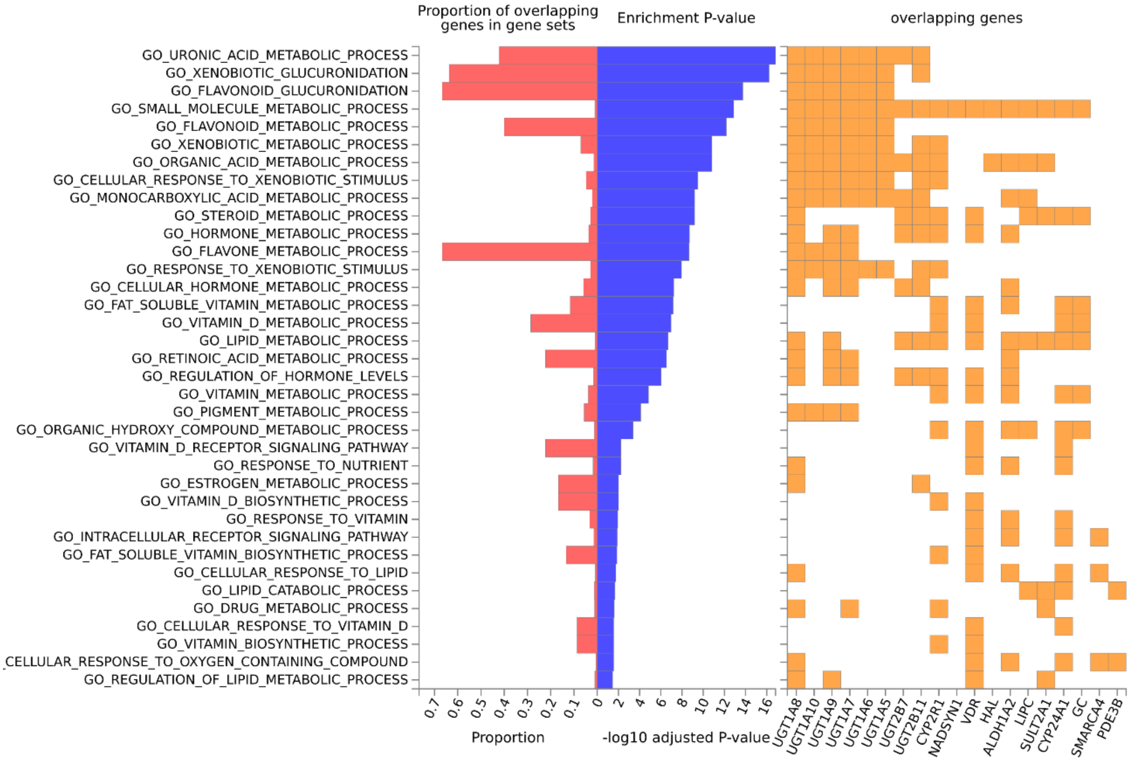

Figure S11. Enrichment analysis of VD deficiency and the VDR gene regarding KEGG pathways (A) and GO in MSigDB on biological processes (B). Pathways and processes that are overrepresented in the gene set of interest are shown. Also, only genes present in a given pathway or process are given. Overrepresentation of the gene set of interest (input genes) regarding pathways and functions was tested using the hypergeometric test against pre-defined gene sets (background genes) obtained from MsigDB or KEGG. Input genes that are overlapping in the pathway or process, the enrichment P-value and the proportion of overlapping genes (input genes relative to the tested gene set) are shown. Source: FUMA
